# Characterisation and correlates of stunting among Malaysian children and adolescents aged 6–19 years

**DOI:** 10.1017/gheg.2019.1

**Published:** 2019-03-04

**Authors:** Uttara Partap, Elizabeth H. Young, Pascale Allotey, Manjinder S. Sandhu, Daniel D. Reidpath

**Affiliations:** 1 Department of Medicine, University of Cambridge, Cambridge, UK; 2 Wellcome Sanger Institute, Hinxton, UK; 3 United Nations University International Institute of Global Health (UNU-IIGH), Kuala Lumpur, Malaysia; 4 Jeffrey Cheah School of Medicine and Health Sciences, Monash University Malaysia, Selangor, Malaysia; 5 South East Asia Community Observatory, Segamat, Malaysia

**Keywords:** Child nutrition, child stunting, health and demographic surveillance, South East Asia, water and sanitation

## Abstract

**Background:**

Despite emerging evidence regarding the reversibility of stunting at older ages, most stunting research continues to focus on children below 5 years of age. We aimed to assess stunting prevalence and examine the sociodemographic distribution of stunting risk among older children and adolescents in a Malaysian population.

**Methods:**

We used cross-sectional data on 6759 children and adolescents aged 6–19 years living in Segamat, Malaysia. We compared prevalence estimates for stunting defined using the Centers for Disease Control and Prevention (CDC) and the World Health Organization (WHO) references, using Cohen's *κ* coefficient. Associations between sociodemographic indices and stunting risk were examined using mixed-effects Poisson regression with robust standard errors.

**Results:**

The classification of children and adolescents as stunted or normal height differed considerably between the two references (CDC *v*. WHO; *κ* for agreement: 0.73), but prevalence of stunting was high regardless of reference (crude prevalence: CDC 29.2%; WHO: 19.1%). Stunting risk was approximately 19% higher among underweight *v*. normal weight children and adolescents (*p* = 0.030) and 21% lower among overweight children and adolescents (*p* = 0.001), and decreased strongly with improved household drinking water sources [risk ratio (RR) for water piped into house: 0.35, 95% confidence interval (95% CI) 0.30–0.41, *p* < 0.001). Protective effects were also observed for improved sanitation facilities (RR for flush toilet: 0.41, 95% CI 0.19–0.88, *p* = 0.023). Associations were not materially affected in multiple sensitivity analyses.

**Conclusions:**

Our findings justify a framework for strategies addressing stunting across childhood, and highlight the need for consensus on a single definition of stunting in older children and adolescents to streamline monitoring efforts.

## Introduction

Notable progress has been made in the reduction of child undernutrition in recent decades. However, child stunting remains an important global health issue [1]. In 2014, 157 million out of 667 million children under the age of 5 years were estimated to be stunted, over half of whom were in Asia [1]. Understood to be a direct result of poor nutrition and chronic infection, stunting is associated with a number of later life adverse outcomes, including cognitive impairment, lower educational achievement, adverse pregnancy outcomes among women and increased cardiometabolic disease risk [2–6]. Given its high burden and far-reaching consequences, comprehensive and well-informed strategies addressing stunting must remain a priority [6–8].

Until recently, stunting was generally understood to occur during the first 1000 days of life, with little potential for recovery in later years [4, 6]. Most research and programmes to date addressing stunting and associated risk factors have thus focused on children up to 5 years of age [9–20]. However, a growing body of evidence from diverse populations indicates the potential to transition between stunted and non-stunted status up till at least 15 years of age [21–29]. Recent research also suggests cognitive gains among children and adolescents who show catch-up growth to normal height at older ages, as well as deficits among older children and adolescents who transition to being stunted [24–28]. More data are needed to better characterise linear growth trajectories and stunting throughout childhood, assess influences on the same, and understand subsequent later life outcomes. This includes a clearer assessment of potential differences in stunting classification by growth reference, given the implications this may have in terms of comparing estimates of stunting burden across populations and informing resource planning for strategies addressing stunting [30], and an exploration of potential determinants. Such evidence would facilitate a more comprehensive understanding of the risk of stunting and potential for recovery across childhood, and contribute to a clearer picture of implications for population health and productivity. We sought to address the current gap in understanding of stunting among older children and adolescents by assessing the prevalence and sociodemographic correlates of stunting in 6759 children and adolescents aged 6–19 years in Segamat, Malaysia.

## Subjects and methods

### Study population and measures of interest

We used data from two rounds of enumeration (2012–2013 and 2013–2014) and a cross-sectional health survey (2013–2014) conducted by the South East Asia Community Observatory health and demographic surveillance system (SEACO HDSS) operating in Segamat, Malaysia [31, 32]. Enumerations included all consenting individuals living within the area covered by SEACO, whilst the health survey collected measures from individuals aged 6 years and above using standardised tools; information was linked across databases [33–37]. Ethical approval for all data collections was obtained from the Monash University Human Research Ethics Committee, and informed consent was obtained from all participants prior to data collection.

Analyses included all children and adolescents with information on age, sex, ethnicity and height (*N* = 6759). Children were defined as individuals aged <10 years, whilst adolescents were defined as individuals aged 10–19 years [38–41]. Information from the 2012–2013 enumeration was used to match children and adolescents to their parents and determine birth order. Data from both enumerations were used to identify the child's household indices including number of household members, bedrooms, bathrooms and living areas, type of toilet, whether the toilet was shared with other households, main source of drinking water and main method of garbage disposal. All other information was obtained from the health survey. This included the child's age, sex and ethnicity, the child's and parents’ current height and weight (measured using a Transtek digital weighing scale with height gauge, model GBS-721), parents’ age, and parents’ and household head's education.

### Variable transformation

We used the World Health Organization (WHO) 2007 and the Centers for Disease Control and Prevention (CDC) 2000 child growth references to express height-for-age *z*-score and classify stunting among children and adolescents [41, 42]. These references are distinct in terms of the reference populations chosen and methods used to derive height-for-age reference curves across childhood and adolescence, and in terms of their recommended cut-offs to define stunting [41–43]. Conversion of absolute height to reference-specific height was done using the Zanthro package in Stata [44]. The WHO 2007 reference classifies stunting as height <−2 standard deviations (s.d.) from the median [41], whilst the CDC 2000 reference classifies stunting as height <5^th^ percentile [42, 45]. Body mass index (BMI) was calculated as weight (kg) divided by the square of the height (m), and BMI-for-age status was also expressed relative to the WHO and CDC references [41, 42, 45] using the Zanthro package [44]. The WHO 2007 reference classifies underweight, normal BMI, overweight and obesity as BMI-for-age <−2 s.d., −2 to 2 s.d., >2 to 3 s.d. and >3 s.d., respectively [41]. The CDC 2000 reference defines underweight, normal BMI, overweight and obesity as <5^th^ percentile, 5^th^ to 84^th^ percentile, 85^th^ to 94^th^ percentile, and ⩾95^th^ percentile, respectively [42, 45]. Maternal BMI was calculated as described above, and maternal underweight was defined as BMI <18.5 kg/m^2^. Parental height was assessed as a continuous variable and also categorised into five groups, as has been done previously (160 and above, 155–159, 150–154, 145–149 and <145 cm) [12]. The number of bedrooms, bathrooms and living areas in the household was divided by the number of members to obtain the ratio of rooms to people.

### Statistical analysis

We first examined crude stunting prevalence obtained with stunting classified using the WHO *v*. the CDC reference in the full study population (*N* = 6759). Prevalence was calculated overall and across demographic subgroups (sex, age and ethnicity). We used Cohen's *κ* statistic to assess the agreement in classification of stunting between the WHO and CDC references, overall and across demographic subgroups. *κ* < 0.6 was defined as poor agreement, 0.6 to <0.8 as moderate agreement, 0.8 to <0.9 as good agreement and ⩾0.9 as excellent agreement [46]. We examined within-household and within-sub-district clustering of stunting in the full population using both references. We then compared adjusted estimates for stunting prevalence using the WHO and CDC references, overall and across demographic subgroups, generated using mixed-effects Poisson regression adjusted for sex, age and ethnicity, and for clustering at the household level.

We then examined associations between stunting and a range of sociodemographic indices in a subset of the population with complete information on all measures of interest, including individual (age, sex, ethnicity, birth order and BMI-for-age status), maternal (height and current underweight) and household measures (bedrooms, bathrooms and living areas per household member, type of toilet, sharing of toilet with other households, main source of drinking water and main method of garbage disposal) (*N* = 3791). Initially, we assessed differences in measures of interest between children and adolescents included in regressions *v*. those who were excluded. Following this, we examined sociodemographic measures of interest across categories of stunting among included children and adolescents. Differences in distributions of measures by inclusion status or by stunting status were assessed using Pearson's χ^2^ tests for categorical variables (Fisher's exact tests for cell counts less than five) and Student's *t*-test for continuous variables. We then examined univariable associations between each measure and stunting (using Poisson regression). The linearity of continuous measures was assessed by comparing models with continuous *v*. categorised measures using likelihood ratio tests.

Following this, we used mixed-effects Poisson regression models with robust standard errors to assess the relation between sociodemographic indices and stunting expressed using the CDC reference. Regression models were built with progressive adjustment for groups of variables, starting with individual measures, and then additionally including maternal and then household measures. Fully-adjusted models included all individual, maternal and household variables described above, and were additionally adjusted for clustering at the household level. To confirm trends and obtain more concise estimates of risk, we additionally examined models (i) including maternal height as a continuous variable, and (ii) including variables with collapsed categories for maternal height, type of toilet and main source of drinking water.

Effect modification of stunting risk by sex was explored by comparing minimally-adjusted mixed-effects Poisson regression models (age, ethnicity, sex, measure of interest) with those including an additional term for interaction (sex × measure of interest) using likelihood ratio tests. Furthermore, we performed a number of sensitivity analyses to assess the robustness of associations observed. Crude and fully adjusted associations between maternal age and stunting were assessed, and the effect of its inclusion on other associations was examined. We similarly examined education of the mother, father and head of household, and paternal height. We assessed the specificity of associations by checking the effect of inclusion of the following orthogonal variables in regression models: maternal heart rate, maternal diastolic blood pressure, availability of Internet from the provider Streamyx in the household, and the ratio of motorcycles in the household to the number of household members. Analysis of complete records was undertaken for these sensitivity analyses.

Additionally, we examined the relation between stunting risk and the co-occurrence of associated sociodemographic indices identified from the preceding regression models. These indices were binarised to facilitate clearer interpretation, and included Malay ethnicity, child underweight, maternal height <145 cm, living in a household with an unprotected water source, living in a household with a bucket, hanging or no latrine, and living in a household with a shared toilet. Mixed-effects Poisson regression with robust standard errors was undertaken with stunting as the outcome and the number of associated indices as the primary exposure. The final model was adjusted for all other sociodemographic indices of interest, and for clustering at the household level.

Finally, we checked associations observed using mixed-effects logistic regression models with robust standard errors, and with mixed-effects linear regression models; final models were built identically to Poisson regression models above. We also ran all analyses using the WHO reference to express height-for-age and classify stunting, to assess comparability in the direction and effect size of estimates of association.

All analyses were performed using Stata 14 (Statacorp, Texas, USA).

## Results

Analyses covered a total of 6759 children and adolescents (49.3% boys). Children aged 6–9 years comprised 26.2% of the population, and approximately 37% each were aged 10–14 and 15–19 years. The majority of children and adolescents were of Malay ethnicity, followed by Chinese and then Indian ethnicity (67.3, 19.9 and 9.8%, respectively). There was no difference in age or ethnicity distributions by sex (Table 1).

**Table 1.** Demographic characteristics of study population

	Overall		Boys		Girls		*p*
*N* (%)	6759		3335	(49.3)	3424	(50.7)	
Age (years)							
6–9	1772	(26.2)	899	(27.0)	873	(25.5)	
10–14	2519	(37.3)	1257	(37.7)	1262	(36.9)	
15–19	2468	(36.5)	1179	(35.4)	1289	(37.6)	0.127
Ethnicity, *n* (%)							
Malay	4548	(67.3)	2246	(67.3)	2302	(67.2)	
Indian	662	(9.8)	332	(10.0)	330	(9.6)	
Chinese	1344	(19.9)	658	(19.7)	686	(20.0)	
Bumiputera/Orang Asli	122	(1.8)	61	(1.8)	61	(1.8)	
Other	83	(1.2)	38	(1.1)	45	(1.3)	0.952

Differences in distributions across categories between girls and boys were compared using Pearson's χ^2^ test.

We first assessed the agreement between the CDC and WHO references in stunting classification and subsequent estimation of prevalence. Stunting prevalence was notably higher when using the CDC reference compared with the WHO reference, with only moderate agreement between the two (crude prevalence using WHO reference: 19.1%, using CDC reference: 29.2%, *κ* for agreement: 0.73) (Table 2, online Supplementary Table S1). Differences in prevalence estimates, and demographic patterns in prevalence, were generally consistent between references (Table 2, online Supplementary Table S1). Stunting was strongly clustered within households, regardless of the reference used (online Supplementary Table S2).

**Table 2.** Prevalence of stunting amongst children in Segamat, Malaysia according to the World Health Organization 2007 *v*. Centers for Disease Control 2000 reference

	WHO		CDC		Agreement^a^
Overall	16.5	(15.1, 17.9)	29.1	(27.8, 30.4)	0.73
Sex, *n* (%)					
Male	19.9	(17.7, 22.2)	28.8	(27.0, 30.6)	0.77
Female	17.9	(15.7, 20.0)	29.4	(27.5, 31.2)	0.69
Age, years, *n* (%)					
6–9	12.9	(11.3, 14.6)	20.1	(18.0, 22.2)	0.75
10–14	18.4	(15.8, 21.0)	26.9	(24.9, 28.9)	0.77
15–19	23.7	(21.8, 25.7)	37.8	(35.4, 40.3)	0.68
Ethnicity, *n* (%)					
Malay	18.1	(16.4, 19.7)	31.8	(30.2, 33.5)	0.72
Indian	13.1	(10.3, 15.9)	24.1	(20.3, 27.8)	0.73
Chinese	12.8	(10.7, 14.9)	22.3	(19.7, 24.8)	0.75
Bumiputera/Orang Asli	19.2	(11.4, 27.0)	34.4	(24.0, 44.8)	0.70
Other	13.1	(5.5, 20.7)	18.7	(9.3, 28.2)	0.91

WHO, World Health Organization; CDC, Centers for Disease Control.

Estimates are for adjusted prevalence, based on mixed-effects Poisson regression models including sex, age and ethnicity and adjusted for clustering at the household level.

a Agreement in classification of stunting between references was calculated using Cohen's *κ* coefficient.

We further explored the relation between stunting and a range of sociodemographic indices in a subset of the population with complete information on all measures (*N* = 3791). Children and adolescents included in this subset were generally comparable to those who were excluded in terms of measures of interest (online Supplementary Table S3). Similar to the full study population, 28.5% of children and adolescents were stunted (CDC reference) (Table 3). Compared with children and adolescents of normal height, stunted children and adolescents were marginally older, and were less commonly of Chinese or Indian ethnicity, overweight or obese, of birth order four or above, and with mothers of height 150 cm or above (*p* < 0.001 for all). Stunted children and adolescents were also less likely to be living in a household with a flush toilet or a toilet not shared with other households (*p* = 0.009 and *p* = 0.025, respectively), or a household where the main source of drinking water was piped into the yard or house (*p* < 0.001) (Table 3).

**Table 3.** Sociodemographic characteristics of study population across categories of stunting

	Overall		Normal height		Stunted^a^		*p*
*N* (%)	3791		2712	(71.5)	1079	(28.5)	
Sex, *n* (%)							
Male	1870	(49.3)	1372	(50.6)	498	(46.2)	
Female	1921	(50.7)	1340	(49.4)	581	(53.9)	0.014
Age, years, mean (s.d.)	12.6	(3.9)	12.2	(3.8)	13.6	(3.7)	<0.001
Ethnicity, *n* (%)							
Malay	2586	(68.2)	1792	(66.1)	794	(73.6)	
Indian	405	(10.7)	316	(11.7)	89	(8.3)	
Chinese	694	(18.3)	530	(19.5)	164	(15.2)	
Bumiputera/Orang Asli	65	(1.7)	43	(1.6)	22	(2.0)	
Other	41	(1.1)	31	(1.1)	10	(0.9)	<0.001
BMI-for-age status^a^, *n* (%)							
Underweight	232	(6.1)	255	(9.4)	134	(12.4)	
Normal	2460	(64.9)	1712	(63.1)	736	(68.2)	
Overweight or obese	1099	(29.0)	745	(27.5)	209	(19.4)	<0.001
Birth order, *n* (%)							
1	1604	(42.3)	1101	(40.6)	503	(46.6)	
2	1150	(30.3)	821	(30.3)	329	(30.5)	
3	635	(16.8)	481	(17.7)	154	(14.3)	
4**+**	402	(10.6)	309	(11.4)	93	(8.6)	<0.001
Maternal height (cm) category, *n* (%)							
160+	613	(16.2)	462	(17.0)	151	(14.0)	
155–159	886	(23.4)	663	(24.5)	223	(20.7)	
150–154	1204	(31.8)	893	(32.9)	311	(28.8)	
145–149	758	(20.0)	491	(18.1)	267	(24.8)	
<145	330	(8.7)	203	(7.5)	127	(11.8)	<0.001
Maternal current underweight, *n* (%)	92	(2.4)	69	(2.5)	23	(2.1)	0.456
Rooms per household member, mean (s.d.)							
Bedrooms	0.6	(0.3)	0.6	(0.3)	0.6	(0.3)	0.582
Bathrooms	0.4	(0.2)	0.4	(0.2)	0.4	(0.2)	0.785
Living areas^b^	0.3	(0.2)	0.3	(0.2)	0.3	(0.2)	0.607
Type of toilet, *n* (%)							
None, bucket or hanging latrine	10	(0.3)	4	(0.2)	6	(0.6)	
Bore hole toilet^c^	110	(2.9)	67	(2.5)	43	(4.0)	
Pour flush toilet	1017	(26.8)	706	(26.0)	311	(28.8)	
Flush toilet with septic tank	1548	(40.8)	1140	(42.0)	408	(37.8)	
Flush toilet connected with sewerage system	1106	(29.2)	795	(29.3)	311	(28.8)	0.009
Toilet shared with other household, *n* (%)	17	(0.5)	8	(0.3)	9	(0.8)	0.025
Main source of drinking water, *n* (%)							
Unprotected source^d^	3	(0.1)	1	(0.0)	2	(0.2)	
Public standpipe or other protected source^e^	96	(2.5)	57	(2.1)	39	(3.6)	
Piped into yard	133	(3.5)	106	(3.9)	27	(2.5)	
Piped into house	3559	(93.9)	2548	(94.0)	1011	(93.7)	<0.001
Main method of garbage disposal, *n* (%)							
Buried, burned or thrown	1042	(27.5)	746	(27.5)	296	(27.4)	
Collected and thrown for recycling	250	(6.6)	177	(6.5)	73	(6.8)	
Collected irregularly by local authority	133	(3.5)	89	(3.3)	44	(4.1)	
Collected regularly by local authority	2366	(62.4)	1700	(62.7)	666	(61.7)	0.665

BMI, body mass index.

**Fig. 1.**
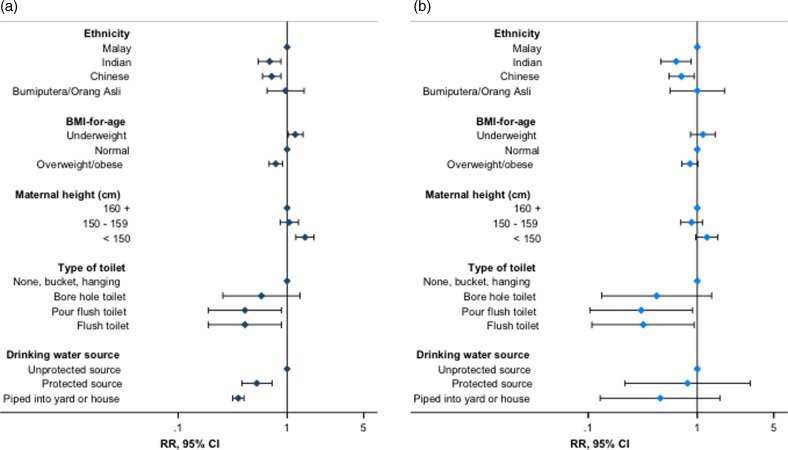
Association of stunting with selected individual, parental and household water and sanitation indices – stunting expressed using the (*a*) Centers for Disease Control and Prevention 2000 reference and (*b*) World Health Organization 2007 reference. Estimates presented from Poisson regression models described in online Supplementary Tables S5 and S10. RR, risk ratio; 95% CI, 95% confidence interval (error bars). Flush toilet includes that connected with septic tank or sewerage system; unprotected drinking water sources: unprotected dug well, or water taken directly from pond or stream; protected sources: public standpipe, protected dug well or spring, or water from bottles or tanker truck.

**Fig. 2.**
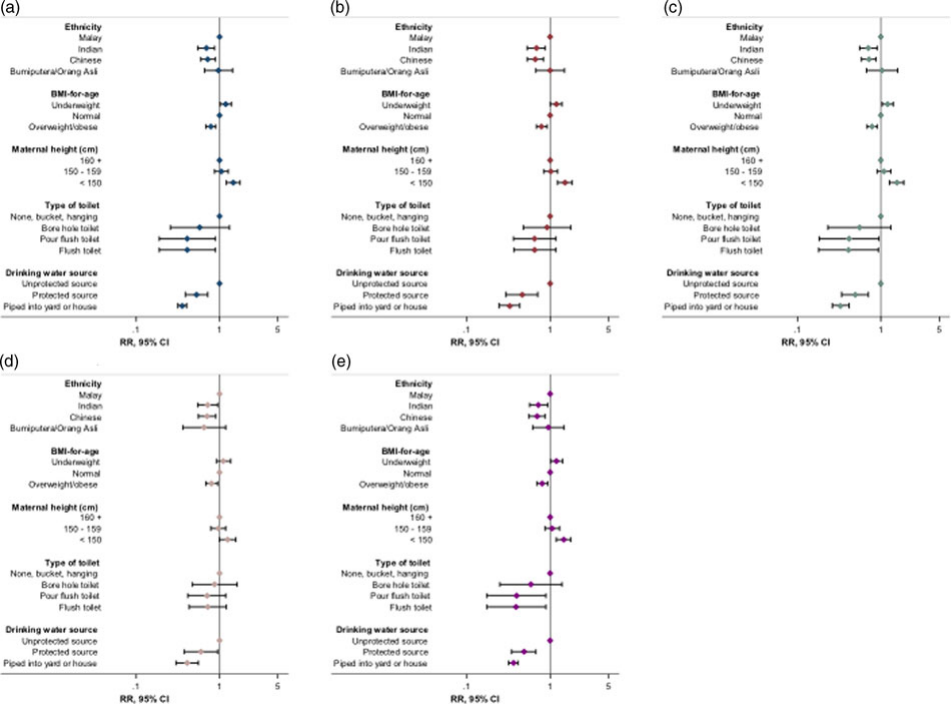
Summary of effect of sensitivity analyses on associations between key indices and stunting risk. (*a*) Original regression model (online Supplementary Table S5; *N* = 3791); (*b*) model with maternal age included (*N* = 3337); (*c*) model with maternal education included (*N* = 3625); (*d*) model with paternal height included (*N* = 2723); (*e*) model with orthogonal variables included (*N* = 3434). RR, risk ratio; 95% CI, 95% confidence interval (error bars); BMI, body mass index. Stunting and BMI-for-age were defined using the Centers for Disease Control and Prevention 2000 reference. Flush toilet includes that connected with septic tank or sewerage system; unprotected drinking water sources: unprotected dug well, or water taken directly from pond or stream; protected sources: public standpipe, protected dug well or spring, or water from bottles or tanker truck. Orthogonal variables: maternal heart rate, maternal diastolic blood pressure, household Streamyx Internet and motorcycles per household member.

**Fig. 3.**
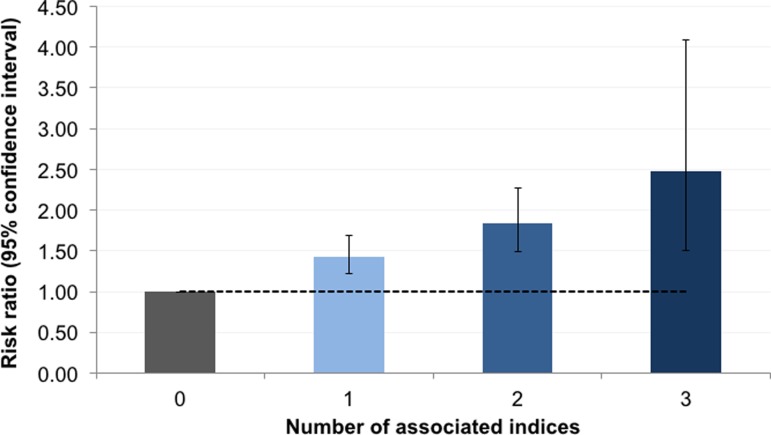
Relative risk of stunting associated with the number of co-occurring risk factors. Stunting was classified using the Centers for Disease Control and Prevention 2000 reference. Dashed line represents null association (relative risk of 1); error bars denote 95% confidence interval. Risk factors included: Malay ethnicity, child underweight, maternal height <145 cm, living in a household with unprotected drinking water source (unprotected dug well, or water taken directly from pond or stream), living in household with no bucket or hanging latrine and living in a household with a shared toilet. Estimates are based on Poisson regression models adjusted for the child's age, sex and body mass index-for-age status, birth order, maternal current underweight, rooms per household member, household's main method of garbage disposal and for clustering at the household level.

Descriptive analyses presented for a subset of children with complete information on all sociodemographic variables of interest above (*N* = 3791), whose data were used for regression analyses (Table 4, Figs 1–3).

Differences in distributions across categories between normal height and stunted were compared using Pearson's χ^2^ test, or Fisher's exact test for variables with cell frequencies <5.

a Stunting and BMI-for-age were classified using the Centers for Disease Control 2000 reference.

b Living areas include dining rooms but not kitchens.

c Bore hole toilet both with or without cover.

d Unprotected sources: unprotected dug well, or water taken directly from pond or stream.

e Protected sources: protected dug well or spring, or water from bottles or tanker truck.

We examined associations between sociodemographic indices and child stunting (CDC reference) using mixed-effects Poisson regression. In fully adjusted analyses, stunting risk increased by 8% per year increase in age [fully adjusted risk ratio (RR): 1.08, 95% confidence interval (95% CI) 1.06–1.09, *p* < 0.001). Children and adolescents of Indian or Chinese ethnicity had an approximately 30% decreased risk of stunting compared with those of Malay ethnicity (RR for Indian ethnicity: 0.66, 95% CI 0.52–0.83, *p* < 0.001; for Chinese ethnicity: 0.68, 95% CI 0.55–0.83, *p* < 0.001) (Table 4, Fig. 1, online Supplementary Table S4). Overweight children and adolescents also had a reduced risk of stunting compared with those of normal BMI, whilst underweight was positively associated with stunting risk (RR for overweight: 0.79, 95% CI 0.69–0.90, *p* = 0.001; for underweight: 1.19, 95% CI 1.02–1.39, *p* = 0.030). Children and adolescents with mothers of height <145 cm had a 53% increased risk of stunting compared with those whose mothers were 160 cm or taller (RR 1.53, 95% CI 1.21–1.92, *p* < 0.001). We found no evidence of associations between sex, birth order or current maternal underweight and child stunting risk (Table 4).

**Table 4.** Relative risk of stunting^a^ associated with sociodemographic indices

	Risk ratio (95% confidence interval)		*p*
Female sex (*v*. male)	1.10	(1.00–1.22)	0.057
Age	1.08	(1.06–1.09)	<0.001
Ethnicity			
Malay	1.00		
Indian	0.66	(0.52–0.83)	<0.001
Chinese	0.68	(0.55–0.83)	<0.001
Bumiputera/Orang Asli	0.96	(0.65–1.40)	0.818
Other	0.91	(0.42–1.93)	0.796
BMI-for-age^a^ status			
Underweight	1.19	(1.02–1.39)	0.030
Normal	1.00		
Overweight or obese	0.79	(0.69–0.90)	0.001
Birth order			
1	1.00		
2	1.03	(0.92–1.14)	0.628
3	0.99	(0.84–1.17)	0.936
4+	1.05	(0.83–1.31)	0.704
Maternal height (cm) category			
160+	1.00		
155–159	1.03	(0.83–1.28)	0.765
150–154	1.06	(0.87–1.29)	0.586
145–149	1.44	(1.18–1.76)	<0.001
<145	1.53	(1.21–1.92)	<0.001
Maternal current underweight (*v*. BMI ⩾18.5 kg/m^2^)	0.86	(0.58–1.27)	0.440
Rooms per household member			
Bedrooms	1.04	(0.79–1.36)	0.787
Bathrooms	0.67	(0.46–0.98)	0.036
Living areas^b^	1.16	(0.73–1.85)	0.519
Type of toilet			
None, bucket or hanging latrine	1.00		
Bore hole toilet^c^	0.57	(0.25–1.30)	0.182
Pour flush toilet	0.41	(0.18–0.89)	0.025
Flush toilet with septic tank	0.38	(0.18–0.84)	0.017
Flush toilet connected with sewerage system	0.46	(0.21–1.02)	0.055
Toilet shared with other household (*v*. not shared)	1.72	(1.07–2.74)	0.025
Main source of drinking water			
Unprotected source^d^	1.00		
Public standpipe or other protected source^e^	0.52	(0.38–0.73)	<0.001
Piped into yard	0.25	(0.15–0.39)	<0.001
Piped into house	0.35	(0.30–0.41)	<0.001
Main method of garbage disposal			
Buried, burned or thrown	1.00		
Collected and thrown for recycling	1.06	(0.84–1.34)	0.603
Collected irregularly by local authority	1.39	(1.02–1.89)	0.036
Collected regularly by local authority	1.22	(1.05–1.41)	0.011

BMI, body mass index.

Estimates based on mixed-effects Poisson regression models adjusted for all other variables above, and for clustering at the household level.

a Stunting and BMI-for-age were classified using the Centers for Disease Control 2000 reference.

b Living areas include dining rooms but not kitchens.

c Bore hole toilet both with or without cover.

d Unprotected sources: unprotected dug well, or water taken directly from pond or stream.

e Protected sources: protected dug well or spring, or water from bottles or tanker truck.

Additionally, children and adolescents whose main source of drinking water was pumped into the house or yard had an approximately 70% reduced risk of stunting *v*. those with access to unprotected sources, with smaller inverse associations for other protected water sources (RR for water piped into yard: 0.25, 95% CI 0.15–0.39, *p* < 0.001; for other protected sources: 0.52, 95% CI 0.38–0.73, *p* < 0.001). Furthermore, children and adolescents with a flush toilet in the household had an approximately 60% decreased risk of stunting *v*. those with a bucket, hanging or no latrine (RR for flush toilet with septic tank: 0.38, 95% CI 0.18–0.84, *p* = 0.017) (Table 4, Fig. 1, online Supplementary Table S4). Consistent trends were observed for related sanitation indices (RR for shared household toilets *v*. not shared: 1.72, 95% CI 1.07–2.74, *p* = 0.025; RR per unit increase in bathrooms per household member: 0.67, 95% CI 0.46–0.98, *p* = 0.036, respectively) (Table 4). We found no evidence of effect modification of associations between measures of interest and stunting risk by sex (online Supplementary Table S6).

We confirmed the robustness and specificity of observed associations through multiple additional checks and sensitivity analyses. Similar magnitudes and directions of association were observed when analyses were repeated with height-for-age and stunting expressed according to the WHO reference (Fig. 1, online Supplementary Table S7). The additional inclusion of maternal age, paternal height, or parental or household head's education to fully adjusted models did not materially change observed associations (Fig. 2, online Supplementary Tables S8–S11), with no evidence of association between parental or head of household's education and stunting risk (online Supplementary Table S9). Furthermore, we observed no association between stunting and theoretically uncorrelated maternal and household variables in fully adjusted regression models, with no effect of their inclusion on other associations (Fig. 2, online Supplementary Table S12). Trends in associations when using linear or logistic regression were consistent with those observed for Poisson regression models (online Supplementary Table S5, data not shown for logistic regression).

We further examined the relation between stunting and the co-occurrence of associated sociodemographic indices identified in the previous regressions. Indices included Malay ethnicity, child underweight, maternal height <145 cm, unprotected drinking water source, none or hanging or bucket latrine in the household, and shared household toilet. Thirty-four (0.9%) children and adolescents had a maximum of three of any of these measures (online Supplementary Table S13). The risk of stunting increased consistently with the number of co-occurring indices, with a greater than twofold increased risk among children and adolescents with three indices *v*. none (RR 2.48, 95% CI 1.51–4.09, *p* < 0.001) (Fig. 3, online Supplementary Table S14).

## Discussion

In this large population of Malaysian children and adolescents, we observed a high burden of stunting, with notable discrepancies in prevalence estimated using two international height references. Stunting was independently associated with indices related to nutritional deficits and poor household environment; associations were robust to a series of sensitivity analyses and to the height reference used. The risk of stunting increased with the number of co-occurring indices. Our results provide strong support for a framework for strategies addressing stunting across childhood, and highlight the need for consensus on a single definition of stunting among older children and adolescents to streamline monitoring efforts.

Evidence on the burden and determinants of stunting among children and adolescents aged over 5 years is limited [22, 47–52]. This includes research on the comparability of height references in the classification of stunting and subsequent estimation of prevalence. We identified only three other studies comparing the WHO and CDC references among older children and adolescents, based on smaller populations of narrower age ranges than in this study [47–49]. All reported discrepancies in stunting classification by reference, although smaller than those observed here [47–49]. The large differences in prevalence estimates by reference in our study population have important implications for decisions relating to targeting of interventions and health resource planning. Furthermore, our results indicate a fundamental gap in the current understanding of normal and compromised linear growth at older ages, including whether this is distinct to population. Further longitudinal research is required in this area, along with a more immediate consensus on the universal use of one reference to harmonise monitoring of stunting in this older age group across populations.

The associations between stunting and environmental and nutritional indices observed in our study are consistent with previous work, which has mainly been undertaken in younger children [9–20]. Evidence from older children and adolescents is scarce, generally based on pre-adolescents and on smaller populations than examined here, with limited exploration of environmental or nutritional measures [22, 50–52]. Among younger children, poor sanitation and drinking water facilities are understood to contribute to stunting through increased risk of acute and chronic enteric infections, leading to malabsorption of nutrients essential to growth [6, 53]. Measures such as underweight or wasting are established indicators of undernutrition resulting from insufficient nutrient intake or absorption, and are thought to at least partly share biological pathways leading to stunting [6, 54]. Our results suggest that poor environment and nutrition are also important determinants of linear growth and stunting in later childhood. Importantly, our study indicates that these indices contribute to stunting risk independently from the potential genetic, intrauterine and shared environmental influences of both parents’ height [55–62]. Targeting these modifiable factors throughout childhood may thus be a useful approach to improving linear growth in this population, with potential for further subsequent intergenerational benefits. On the other hand, the implications of the increased risk of stunting observed among Malay children and adolescents are unclear. There has been debate regarding whether ethnicity-specific differences observed in child growth beyond 5 years of age may be a result of modifiable environmental exposures or genetic differences in growth potential [41]. Whilst we accounted for potential differences in household environment and BMI in this study, we were unable to account for other ethnicity-specific factors such as diet that may additionally explain such differences in risk. Our observations point to an important area of inquiry that may be best addressed through additional studies, including qualitative or genetic research.

Certain observations in our study stand somewhat in contrast to the totality of previous evidence on determinants of stunting risk. Measures of overcrowding and family size [17, 51, 52], maternal age and underweight [12, 15, 18, 56], and parental education [6, 10, 11, 14–16, 19] or related measures of socioeconomic status [63, 64] have previously been implicated in poor linear growth, but were not associated with stunting here. As has been observed with risk factors in other studies, it may be that these measures are not important determinants of stunting in our study population [9, 10, 15, 50]. Importantly, we observed increased stunting risk with a low bathroom to household member ratio, and with sharing of toilets between households, suggesting that overcrowding may be relevant in more extreme contexts in this population. As such, these results reiterate the importance of population-specific research to inform suitable strategies addressing stunting.

Valuable insights for the design and targeting of interventions may be gained by examining the co-occurrence of risk factors and their collective effect on stunting risk. Yet, to our knowledge, this has not been explored in earlier studies. In our study population, out of six measures associated with stunting, a small group of children and adolescents had the maximum of three co-occurring indices, and stunting risk increased generally consistently with the number of co-occurring indices. Our population size limited our ability to assess in greater detail the risk of stunting associated with specific combinations of indices, which could be more informative for the purposes of intervention targeting. Nonetheless, we highlight here a potentially useful approach to identify sub-populations at greater risk of stunting through examining the extent and patterns of distribution of co-occurring risk factors.

Child undernutrition is being increasingly recognised as a public health issue of concern in Malaysia, with recent estimates suggesting a notable and rising burden of stunting among children under 5 years of age [65]. Both governmental and non-governmental organisations have acknowledged the growing need to implement targeted strategies to address undernutrition among children in Malaysia [65–67]. Importantly, these would have to be undertaken in coordination with interventions addressing the simultaneously increasing burden of overweight and obesity among children [66, 68–72]. Given the high overweight and obesity prevalence observed among children and adolescents in our study population, our results provide further support for the need for a continued research and programmatic focus on the double burden of malnutrition throughout childhood in Malaysia.

To our knowledge, ours is among the largest of the few studies to date that have examined the prevalence and determinants of stunting in children and adolescents older than 5 years of age [22, 47–52]. Our study provides strong evidence for associations between environmental and nutritional indices and stunting risk: observed associations were specific, robust to adjustment for multiple covariates and independent of the height reference used to express stunting. For household sanitation, we assessed three measures capturing distinct aspects of the availability and quality of facilities, all of which were independently and consistently related to stunting risk. As this is a cross-sectional analysis, we are unable to make definitive statements regarding causality, and to more clearly distinguish between the relative contribution to stunting risk of current *v*. historical nutritional and environmental exposures, which may be correlated [50]. Furthermore, we were unable to consider more detailed measures capturing dimensions relating to nutrition, such as diet or food security. However, in light of the previous research suggesting the potential for transition between stunted and non-stunted status at older ages [21–29], we believe our study provides key information regarding potential factors that may inform such transitions. Importantly, our study indicates that these are no different from factors influencing linear growth at younger ages, suggesting that broadening the current conceptual view of stunting to one that extends across childhood may ultimately allow for greater public health gains.

To conclude, we report independent associations between stunting and nutritional and household environmental indices in this population of older children and adolescents from Malaysia, with notable increases in risk associated with the co-occurrence of indices. Importantly, we observed a high burden of stunting in this population, with large differences in stunting classification by height reference. In the context of the increasing prevalence of overweight and obesity in this and similar populations [71, 73], our data on stunting point to the complex impact of malnutrition. Our study supports a framework for stunting across childhood, and highlights the need for consensus on a single height reference to monitor stunting at older ages. It provides a basis for further longitudinal research on the exact definition, determinants and consequences of linear growth and stunting throughout childhood in diverse populations. Such work will be fundamental to the design of strategies that may more comprehensively and effectively address childhood stunting worldwide.

## Supplementary material

For supplementary material accompanying this paper visit https://doi.org/10.1017/gheg.2019.1. click here to view supplementary material
