# Supplementary material for: Characterisation and correlates of stunting among Malaysian children and adolescents aged 6–19 years
Source: Glob Health Epidemiol Genom. 2019 Mar 4;4:e2. doi: 10.1017/gheg.2019.1 (PMC6415126; doi:10.1017/gheg.2019.1)
Supplement: Supplementary file 1 [file S2054420019000010sup001.docx]

**Characterisation and correlates of stunting in a Malaysian children aged 6-19 years**

**Online supplemental material**

| **Table S1. Distribution of anthropometric indicators among the study population using the WHO 2007 and the CDC 2000 references.** | | | | | | | | | | | |
| --- | --- | --- | --- | --- | --- | --- | --- | --- | --- | --- | --- |
|  |  | **Overall** | |  | **Boys** | |  | **Girls** | |  | ***P*** |
|  |  |  |  |  |  |  |  |  |  |  |  |
| **WHO** |  |  |  |  |  |  |  |  |  |  |  |
|  |  |  |  |  |  |  |  |  |  |  |  |
| **Height-for-age** |  |  |  |  |  |  |  |  |  |  |  |
| **Normal height-for-age** |  | 5465 | (80.9) |  | 2660 | (79.8) |  | 2805 | (81.9) |  |  |
| **Stunted** |  | 1294 | (19.1) |  | 675 | (20.2) |  | 619 | (18.1) |  | 0.024 |
|  |  |  |  |  |  |  |  |  |  |  |  |
| **BMI-for-age** |  |  |  |  |  |  |  |  |  |  |  |
| **Underweight** |  | 390 | (5.8) |  | 194 | (5.8) |  | 196 | (5.7) |  |  |
| **Normal** |  | 4291 | (63.5) |  | 2023 | (60.7) |  | 2268 | (66.2) |  |  |
| **Overweight** |  | 1119 | (16.6) |  | 561 | (16.8) |  | 558 | (16.3) |  |  |
| **Obese** |  | 959 | (14.2) |  | 557 | (16.7) |  | 402 | (11.7) |  | <0.001 |
|  |  |  |  |  |  |  |  |  |  |  |  |
| **Weight-for-age^1^** |  |  |  |  |  |  |  |  |  |  |  |
| **Low weight-for-age** |  | 199 | (9.0) |  | 100 | (8.9) |  | 99 | (9.2) |  |  |
| **Normal weight-for-age** |  | 1721 | (78.2) |  | 854 | (76.0) |  | 867 | (80.4) |  |  |
| **High weight-for-age** |  | 282 | (12.8) |  | 169 | (15.0) |  | 113 | (10.5) |  | 0.006 |
|  |  |  |  |  |  |  |  |  |  |  |  |
| **CDC** |  |  |  |  |  |  |  |  |  |  |  |
|  |  |  |  |  |  |  |  |  |  |  |  |
| **Height-for-age** |  |  |  |  |  |  |  |  |  |  |  |
| **Normal height-for-age** |  | 4788 | (70.8) |  | 2374 | (71.2) |  | 2414 | (70.5) |  |  |
| **Stunted** |  | 1971 | (29.2) |  | 961 | (28.8) |  | 1010 | (29.5) |  | 0.537 |
|  |  |  |  |  |  |  |  |  |  |  |  |
| **BMI-for-age** |  |  |  |  |  |  |  |  |  |  |  |
| **Underweight** |  | 646 | (9.6) |  | 323 | (9.7) |  | 323 | (9.4) |  |  |
| **Normal** |  | 4327 | (64.0) |  | 2044 | (61.3) |  | 2283 | (66.7) |  |  |
| **Overweight** |  | 867 | (12.8) |  | 446 | (13.4) |  | 421 | (12.3) |  |  |
| **Obese** |  | 919 | (13.6) |  | 522 | (15.7) |  | 397 | (11.6) |  | <0.001 |
|  |  |  |  |  |  |  |  |  |  |  |  |
| **Weight-for-age** |  |  |  |  |  |  |  |  |  |  |  |
| **Low weight-for-age** |  | 1179 | (17.4) |  | 586 | (17.6) |  | 593 | (17.3) |  |  |
| **Normal weight-for-age** |  | 5000 | (74.0) |  | 2407 | (72.2) |  | 2593 | (75.7) |  |  |
| **High weight-for-age** |  | 580 | (8.6) |  | 342 | (10.3) |  | 238 | (7.0) |  | <0.001 |
|  |  |  |  |  |  |  |  |  |  |  |  |
| Differences in distributions across categories between males and females were compared using Pearson's Chi squared test. | | | | | | | | | | | |
| ^1^Weight-for-age is calculated only up to and including age 10 years in the WHO 2007 growth reference (N = 2202 in this dataset). | | | | | | | | | | | |

| **Table S2. Clustering of height-for-age and stunting among children in the study population.** | | | | | | | | |
| --- | --- | --- | --- | --- | --- | --- | --- | --- |
|  |  | **Unadjusted intraclass correlation coefficient (95% confidence interval)** | | | | | | |
|  |  | **Household (N = 6680)** | | |  | **Sub-district (N = 4739)** | | |
|  |  |  |  |  |  |  |  |  |
| **CDC reference** |  |  |  |  |  |  |  |  |
| **Height-for-age z-score** |  | 0.39 | (0.36, | 0.42) |  | 0.00 | (0.00, | 0.01) |
| **Stunting** |  | 0.27 | (0.23, | 0.30) |  | 0.00 | (0.00, | 0.01) |
|  |  |  |  |  |  |  |  |  |
| **WHO reference** |  |  |  |  |  |  |  |  |
| **Height-for-age z-score** |  | 0.39 | (0.36, | 0.42) |  | 0.00 | (0.00, | 0.01) |
| **Stunting** |  | 0.29 | (0.25, | 0.32) |  | 0.01 | (0.00, | 0.02) |
|  |  |  |  |  |  |  |  |  |

| **Table S3. Sociodemographic characteristics of population excluded from analyses versus those included.** | | | | | | | | | | |
| --- | --- | --- | --- | --- | --- | --- | --- | --- | --- | --- |
|  |  | **N** |  | **Excluded** | |  | **Included** | |  | ***P*** |
|  |  |  |  |  |  |  |  |  |  |  |
| **N (%)** |  | 6759 |  | 2968 | (43.9) |  | 3791 | (56.1) |  |  |
|  |  |  |  |  |  |  |  |  |  |  |
| **Stunted, n (%)^1^** |  | 6759 |  | 892 | (30.1) |  | 1079 | (28.5) |  | 0.153 |
|  |  |  |  |  |  |  |  |  |  |  |
| **Sex, n (%)** |  | 6759 |  |  |  |  |  |  |  |  |
| **Male** |  |  |  | 1465 | (49.4) |  | 1870 | (49.3) |  |  |
| **Female** |  |  |  | 1503 | (50.6) |  | 1921 | (50.7) |  | 0.979 |
|  |  |  |  |  |  |  |  |  |  |  |
| **Age, years, mean (SD)** |  | 6759 |  | 12.7 | (3.9) |  | 12.6 | (3.9) |  | 0.457 |
|  |  |  |  |  |  |  |  |  |  |  |
| **Ethnicity, n (%)** |  | 6759 |  |  |  |  |  |  |  |  |
| **Malay** |  |  |  | 1962 | (66.1) |  | 2586 | (68.2) |  |  |
| **Indian** |  |  |  | 257 | (8.7) |  | 405 | (10.7) |  |  |
| **Chinese** |  |  |  | 650 | (21.9) |  | 694 | (18.3) |  |  |
| **Bumiputera/Orang Asli** |  |  |  | 57 | (1.9) |  | 65 | (1.7) |  |  |
| **Other** |  |  |  | 42 | (1.4) |  | 41 | (1.1) |  | <0.001 |
|  |  |  |  |  |  |  |  |  |  |  |
| **BMI-for-age^1^, n (%)** |  | 6759 |  |  |  |  |  |  |  |  |
| **Underweight** |  |  |  | 158 | (5.3) |  | 232 | (6.1) |  |  |
| **Normal** |  |  |  | 1831 | (61.7) |  | 2460 | (64.9) |  |  |
| **Overweight or obese** |  |  |  | 979 | (33.0) |  | 1099 | (29.0) |  | 0.001 |
|  |  |  |  |  |  |  |  |  |  |  |
| **Birth order, n (%)** |  | 5821 |  |  |  |  |  |  |  |  |
| **1** |  |  |  | 881 | (43.4) |  | 1604 | (42.3) |  |  |
| **2** |  |  |  | 603 | (29.7) |  | 1150 | (30.3) |  |  |
| **3** |  |  |  | 332 | (16.4) |  | 635 | (16.8) |  |  |
| **4 +** |  |  |  | 214 | (10.5) |  | 402 | (10.6) |  | 0.880 |
|  |  |  |  |  |  |  |  |  |  |  |
| **Maternal height (cm) category, n (%)** |  | 5038 |  |  |  |  |  |  |  |  |
| **160 +** |  |  |  | 260 | (20.9) |  | 613 | (16.2) |  |  |
| **155 - 159** |  |  |  | 282 | (22.6) |  | 886 | (23.4) |  |  |
| **150 - 154** |  |  |  | 372 | (29.8) |  | 1204 | (31.8) |  |  |
| **145-149** |  |  |  | 235 | (18.9) |  | 758 | (20.0) |  |  |
| **< 145** |  |  |  | 98 | (7.9) |  | 330 | (8.7) |  | 0.006 |
|  |  |  |  |  |  |  |  |  |  |  |
| **Maternal current underweight, n (%)** |  | 4985 |  | 31 | (2.6) |  | 92 | (2.4) |  | 0.742 |
|  |  |  |  |  |  |  |  |  |  |  |
| **Rooms per household member, mean (SD)** | |  |  |  |  |  |  |  |  |  |
| **Bedrooms** |  | 5904 |  | 0.6 | (0.3) |  | 0.6 | (0.3) |  | 0.301 |
| **Bathrooms** |  | 5898 |  | 0.4 | (0.2) |  | 0.4 | (0.2) |  | 0.566 |
| **Living areas^2^** |  | 5934 |  | 0.3 | (0.2) |  | 0.3 | (0.2) |  | 0.375 |
|  |  |  |  |  |  |  |  |  |  |  |
| **Type of toilet, n (%)** |  | 4945 |  |  |  |  |  |  |  |  |
| **None, bucket or hanging latrine** |  |  |  | 5 | (0.4) |  | 10 | (0.3) |  |  |
| **Bore hole toilet^3^** |  |  |  | 44 | (3.8) |  | 110 | (2.9) |  |  |
| **Pour flush toilet** |  |  |  | 350 | (30.3) |  | 1017 | (26.8) |  |  |
| **Flush toilet with septic tank** |  |  |  | 472 | (40.9) |  | 1548 | (40.8) |  |  |
| **Flush toilet connected with sewerage system** |  |  |  | 283 | (24.5) |  | 1106 | (29.2) |  | 0.007 |
|  |  |  |  |  |  |  |  |  |  |  |
| **Toilet shared with other household, n (%)** | | 4949 |  | 7 | (0.6) |  | 17 | (0.5) |  | 0.503 |
|  |  |  |  |  |  |  |  |  |  |  |
| **Main source of drinking water, n (%)** |  | 4949 |  |  |  |  |  |  |  |  |
| **Unprotected source^4^** |  |  |  | 2 | (0.2) |  | 3 | (0.1) |  |  |
| **Public standpipe or other protected source^5^** |  |  |  | 19 | (1.6) |  | 96 | (2.5) |  |  |
| **Piped into yard** |  |  |  | 37 | (3.2) |  | 133 | (3.5) |  |  |
| **Piped into house** |  |  |  | 1100 | (95.0) |  | 3559 | (93.9) |  | 0.193 |
|  |  |  |  |  |  |  |  |  |  |  |
| **Main method of garbage disposal, n (%)** |  | 4875 |  |  |  |  |  |  |  |  |
| **Buried, burned or thrown** |  |  |  | 311 | (28.7) |  | 1042 | (27.5) |  |  |
| **Collected and thrown for recycling** |  |  |  | 42 | (3.9) |  | 250 | (6.6) |  |  |
| **Collected irregularly by local authority** |  |  |  | 27 | (2.5) |  | 133 | (3.5) |  |  |
| **Collected regularly by local authority** |  |  |  | 704 | (64.9) |  | 2366 | (62.4) |  | 0.002 |
|  |  |  |  |  |  |  |  |  |  |  |
| Differences in distributions across categories between normal height and stunted were compared using Pearson's Chi squared test, or Fisher's exact test for variables with cell frequencies < 5. | | | | | | | | | | |
| ^1^Stunting and BMI-for-age were classified using the CDC 2000 reference. ^2^Living areas include dining rooms but not kitchens; ^3^Bore hole toilet both with or without cover; ^4^Unprotected sources: unprotected dug well, or water taken directly from pond or stream; ^5^Protected sources: protected dug well or spring, or water from bottles or tanker truck. | | | | | | | | | | |

| **Table S4. Relative risk of stunting^1^ associated with sociodemographic indices (compressed categories).** | | | | | |
| --- | --- | --- | --- | --- | --- |
|  |  | **Risk ratio (95% confidence interval)** | | | ***P*** |
|  |  |  |  |  |  |
| **Female sex (versus male)** |  | 1.10 | (1.00, | 1.22) | 0.052 |
|  |  |  |  |  |  |
| **Age** |  | 1.08 | (1.06, | 1.09) | <0.001 |
|  |  |  |  |  |  |
| **Ethnicity** |  |  |  |  |  |
| **Malay** |  | 1.00 |  |  |  |
| **Indian** |  | 0.69 | (0.54, | 0.88) | 0.002 |
| **Chinese** |  | 0.72 | (0.59, | 0.88) | 0.001 |
| **Bumiputera/Orang Asli** |  | 0.97 | (0.66, | 1.42) | 0.858 |
| **Other** |  | 0.91 | (0.43, | 1.91) | 0.807 |
|  |  |  |  |  |  |
| **BMI-for-age status^1^, n (%)** |  |  |  |  |  |
| **Underweight** |  | 1.19 | (1.02, | 1.39) | 0.030 |
| **Normal** |  | 1.00 |  |  |  |
| **Overweight or obese** |  | 0.79 | (0.68, | 0.90) | 0.001 |
|  |  |  |  |  |  |
| **Birth order** |  |  |  |  |  |
| **1** |  | 1.00 |  |  |  |
| **2** |  | 1.03 | (0.92, | 1.14) | 0.651 |
| **3** |  | 0.99 | (0.84, | 1.16) | 0.876 |
| **4 +** |  | 1.03 | (0.82, | 1.29) | 0.819 |
|  |  |  |  |  |  |
| **Maternal height (cm) category** |  |  |  |  |  |
| **160 +** |  | 1.00 |  |  |  |
| **150 - 159** |  | 1.05 | (0.87, | 1.26) | 0.628 |
| **< 150** |  | 1.45 | (1.20, | 1.75) | <0.001 |
|  |  |  |  |  |  |
| **Maternal current underweight (versus BMI ≥ 18.5)** |  | 0.86 | (0.58, | 1.27) | 0.440 |
|  |  |  |  |  |  |
| **Rooms per household member** |  |  |  |  |  |
| **Bedrooms** |  | 1.08 | (0.83, | 1.41) | 0.563 |
| **Bathrooms** |  | 0.65 | (0.45, | 0.94) | 0.023 |
| **Living areas^2^** |  | 1.07 | (0.68, | 1.70) | 0.770 |
|  |  |  |  |  |  |
| **Type of toilet** |  |  |  |  |  |
| **None, bucket or hanging latrine** |  | 1.00 |  |  |  |
| **Bore hole toilet^3^** |  | 0.58 | (0.26, | 1.31) | 0.188 |
| **Pour flush toilet** |  | 0.41 | (0.19, | 0.89) | 0.023 |
| **Flush toilet with septic tank or sewerage system** |  | 0.41 | (0.19, | 0.88) | 0.023 |
|  |  |  |  |  |  |
| **Toilet shared with other household (versus not shared)** |  | 1.77 | (1.09, | 2.86) | 0.020 |
|  |  |  |  |  |  |
| **Main source of drinking water** |  |  |  |  |  |
| **Unprotected source^4^** |  | 1.00 |  |  |  |
| **Public standpipe or other protected source^5^** |  | 0.53 | (0.39, | 0.73) | <0.001 |
| **Piped into yard or house** |  | 0.36 | (0.32, | 0.40) | <0.001 |
|  |  |  |  |  |  |
| **Main method of garbage disposal** |  |  |  |  |  |
| **Buried, burned or thrown** |  | 1.00 |  |  |  |
| **Collected and thrown for recycling** |  | 1.10 | (0.87, | 1.39) | 0.428 |
| **Collected irregularly by local authority** |  | 1.33 | (0.98, | 1.80) | 0.070 |
| **Collected regularly by local authority** |  | 1.23 | (1.06, | 1.42) | 0.008 |
|  |  |  |  |  |  |
| Estimates based on mixed-effects Poisson regression models adjusted for all other variables above, and for clustering at the household level. | | | | | |
| ^1^Stunting and BMI-for-age were classified using the CDC 2000 reference. ^2^Living areas include dining rooms but not kitchens; ^3^Bore hole toilet both with or without cover; ^4^Unprotected sources: unprotected dug well, or water taken directly from pond or stream; ^5^Protected sources: protected dug well or spring, or water from bottles or tanker truck. | | | | | |

| **Table S5. Associations between height-for-age^1^ and sociodemographic indices.** | | | | | |
| --- | --- | --- | --- | --- | --- |
|  |  | **β (95% confidence interval)** | | | ***P*** |
|  |  |  |  |  |  |
| **Female sex (versus male)** |  | -0.05 | (-0.12, | 0.03) | 0.223 |
|  |  |  |  |  |  |
| **Age** |  | -0.09 | (-0.10, | -0.08) | <0.001 |
|  |  |  |  |  |  |
| **Ethnicity** |  |  |  |  |  |
| **Malay** |  | 1.00 |  |  |  |
| **Indian** |  | 0.32 | (0.16, | 0.49) | <0.001 |
| **Chinese** |  | 0.31 | (0.17, | 0.46) | <0.001 |
| **Bumiputera/Orang Asli** |  | 0.17 | (-0.21, | 0.56) | 0.376 |
| **Other** |  | 0.20 | (-0.21, | 0.61) | 0.338 |
|  |  |  |  |  |  |
| **BMI-for-age status^1^, n (%)** |  |  |  |  |  |
| **Underweight** |  | 0.03 | (-0.09, | 0.16) | 0.599 |
| **Normal** |  | 1.00 |  |  |  |
| **Overweight or obese** |  | 0.32 | (0.23, | 0.41) | <0.001 |
|  |  |  |  |  |  |
| **Birth order** |  |  |  |  |  |
| **1** |  | 1.00 |  |  |  |
| **2** |  | -0.04 | (-0.13, | 0.04) | 0.348 |
| **3** |  | -0.03 | (-0.14, | 0.08) | 0.617 |
| **4 +** |  | -0.09 | (-0.24, | 0.06) | 0.253 |
|  |  |  |  |  |  |
| **Maternal height (cm) category** |  |  |  |  |  |
| **160 +** |  | 1.00 |  |  |  |
| **155 - 159** |  | -0.11 | (-0.26, | 0.05) | 0.168 |
| **150 - 154** |  | -0.19 | (-0.33, | -0.04) | 0.011 |
| **145-149** |  | -0.36 | (-0.51, | -0.20) | <0.001 |
| **< 145** |  | -0.59 | (-0.79, | -0.39) | <0.001 |
|  |  |  |  |  |  |
| **Maternal current underweight (versus BMI ≥ 18.5)** |  | 0.08 | (-0.22, | 0.38) | 0.597 |
|  |  |  |  |  |  |
| **Rooms per household member** |  |  |  |  |  |
| **Bedrooms** |  | -0.02 | (-0.24, | 0.19) | 0.834 |
| **Bathrooms** |  | 0.22 | (-0.10, | 0.53) | 0.174 |
| **Living areas^2^** |  | 0.06 | (-0.31, | 0.43) | 0.750 |
|  |  |  |  |  |  |
| **Type of toilet** |  |  |  |  |  |
| **None, bucket or hanging latrine** |  | 1.00 |  |  |  |
| **Bore hole toilet^3^** |  | 0.57 | (-0.38, | 1.52) | 0.241 |
| **Pour flush toilet** |  | 0.89 | (-0.02, | 1.79) | 0.055 |
| **Flush toilet with septic tank** |  | 0.93 | (0.03, | 1.83) | 0.042 |
| **Flush toilet connected with sewerage system** |  | 0.93 | (0.03, | 1.83) | 0.043 |
|  |  |  |  |  |  |
| **Toilet shared with other household (versus not shared)** |  | -0.67 | (-1.34, | 0.00) | 0.050 |
|  |  |  |  |  |  |
| **Main source of drinking water** |  |  |  |  |  |
| **Unprotected source^4^** |  | 1.00 |  |  |  |
| **Public standpipe or other protected source^5^** |  | 0.36 | (-1.20, | 1.92) | 0.653 |
| **Piped into yard** |  | 0.88 | (-0.67, | 2.43) | 0.267 |
| **Piped into house** |  | 0.66 | (-0.87, | 2.20) | 0.396 |
|  |  |  |  |  |  |
| **Main method of garbage disposal** |  |  |  |  |  |
| **Buried, burned or thrown** |  | 1.00 |  |  |  |
| **Collected and thrown for recycling** |  | 0.05 | (-0.16, | 0.25) | 0.641 |
| **Collected irregularly by local authority** |  | -0.14 | (-0.40, | 0.13) | 0.315 |
| **Collected regularly by local authority** |  | -0.20 | (-0.32, | -0.08) | 0.001 |
|  |  |  |  |  |  |
| Estimates based on mixed-effects linear regression models adjusted for all other variables above, and for clustering at the household level. | | | | | |
| ^1^Height-for-age and BMI-for-age were expressed using the CDC 2000 reference. ^2^Living areas include dining rooms but not kitchens; ^3^Bore hole toilet both with or without cover; ^4^Unprotected sources: unprotected dug well, or water taken directly from pond or stream; ^5^Protected sources: protected dug well or spring, or water from bottles or tanker truck. | | | | | |

| **Table S6. Exploration of interactions between sex and other variables of interest in the study population.** | | | |
| --- | --- | --- | --- |
| **Variable of interest** |  | **N** | ***P^1^*** |
|  |  |  |  |
| **Age** |  | 6680 | 0.075 |
| **Ethnicity** |  | 6680 | 0.144 |
| **BMI-for-age status** |  | 3791 | 0.422 |
| **Birth order** |  | 3791 | 0.966 |
|  |  |  |  |
| **Maternal height (cm) category** |  | 3791 | 0.121 |
| **Maternal current underweight** |  | 3791 | 0.473 |
|  |  |  |  |
| **Bedrooms per household member** |  | 3791 | 0.754 |
| **Bathrooms per household member** |  | 3791 | 0.474 |
| **Living areas per household member** |  | 3791 | 0.544 |
| **Type of toilet** |  | 3791 | 0.705 |
| **Toilet shared with other household** |  | 3791 | 0.624 |
| **Main source of drinking water** |  | 3791 | 0.318 |
| **Main method of garbage disposal** |  | 3791 | 0.571 |
|  |  |  |  |
| Stunting and BMI-for-age were classified using the CDC reference. | | | |
| ^1^*P* for likelihood ratio rest comparing models with and without terms for interaction between child's sex and the variable of interest. | | | |
| Models for age were additionally adjusted for ethnicity, and vice versa. Models for all other variables were additionally adjusted for age and ethnicity, and all models were adjusted for clustering at the household level. | | | |

| **Table S7. Sensitivity analysis: associations between stunting or height-for-age^1^ and sociodemographic indices (WHO reference).** | | | | | | | | | | |
| --- | --- | --- | --- | --- | --- | --- | --- | --- | --- | --- |
|  |  | **Poisson regression** | | | |  | **Linear regression** | | | |
|  |  | **Risk ratio (95% confidence interval)** | | | ***P*** |  | **β (95% confidence interval)** | | | ***P*** |
|  |  |  |  |  |  |  |  |  |  |  |
| **Female sex (versus male)** | | 0.94 | (0.82, | 1.08) | 0.394 |  | -0.03 | (-0.11, | 0.04) | 0.385 |
|  |  |  |  |  |  |  |  |  |  |  |
| **Age** |  | 1.07 | (1.05, | 1.09) | <0.001 |  | -0.09 | (-0.11, | -0.08) | <0.001 |
|  |  |  |  |  |  |  |  |  |  |  |
| **Ethnicity** |  |  |  |  |  |  |  |  |  |  |
| **Malay** |  | 1.00 |  |  |  |  | 1.00 |  |  |  |
| **Indian** |  | 0.62 | (0.45, | 0.85) | 0.003 |  | 0.32 | (0.15, | 0.49) | <0.001 |
| **Chinese** |  | 0.68 | (0.52, | 0.90) | 0.006 |  | 0.33 | (0.18, | 0.47) | <0.001 |
| **Bumiputera/Orang Asli** |  | 0.98 | (0.55, | 1.72) | 0.933 |  | 0.19 | (-0.19, | 0.58) | 0.327 |
| **Other** |  | 1.04 | (0.43, | 2.53) | 0.932 |  | 0.21 | (-0.20, | 0.63) | 0.311 |
|  |  |  |  |  |  |  |  |  |  |  |
| **BMI-for-age^1^ status** |  |  |  |  |  |  |  |  |  |  |
| **Underweight** |  | 1.13 | (0.87, | 1.46) | 0.377 |  | 0.16 | (0.00, | 0.32) | 0.047 |
| **Normal** |  | 1.00 |  |  |  |  | 1.00 |  |  |  |
| **Overweight or obese** |  | 0.85 | (0.72, | 1.01) | 0.060 |  | 0.31 | (0.23, | 0.40) | <0.001 |
|  |  |  |  |  |  |  |  |  |  |  |
| **Birth order** |  |  |  |  |  |  |  |  |  |  |
| **1** |  | 1.00 |  |  |  |  | 1.00 |  |  |  |
| **2** |  | 1.00 | (0.87, | 1.16) | 0.969 |  | -0.04 | (-0.12, | 0.05) | 0.382 |
| **3** |  | 0.91 | (0.73, | 1.14) | 0.407 |  | -0.03 | (-0.15, | 0.08) | 0.590 |
| **4 +** |  | 0.95 | (0.70, | 1.30) | 0.761 |  | -0.10 | (-0.25, | 0.05) | 0.191 |
|  |  |  |  |  |  |  |  |  |  |  |
| **Maternal height (cm) category** | | | |  |  |  |  |  |  |  |
| **160 +** |  | 1.00 |  |  |  |  | 1.00 |  |  |  |
| **155 - 159** |  | 0.90 | (0.69, | 1.18) | 0.449 |  | -0.10 | (-0.25, | 0.05) | 0.203 |
| **150 - 154** |  | 0.88 | (0.69, | 1.12) | 0.306 |  | -0.19 | (-0.33, | -0.04) | 0.011 |
| **145-149** |  | 1.14 | (0.88, | 1.46) | 0.325 |  | -0.36 | (-0.51, | -0.20) | <0.001 |
| **< 145** |  | 1.45 | (1.09, | 1.92) | 0.011 |  | -0.58 | (-0.78, | -0.38) | <0.001 |
|  |  |  |  |  |  |  |  |  |  |  |
| **Maternal current underweight (versus BMI ≥ 18.5)** | | 0.87 | (0.48, | 1.57) | 0.638 |  | 0.06 | (-0.24, | 0.36) | 0.684 |
|  |  |  |  |  |  |  |  |  |  |  |
| **Rooms per household member** | | | |  |  |  |  |  |  |  |
| **Bedrooms** |  | 0.93 | (0.65, | 1.34) | 0.716 |  | -0.03 | (-0.25, | 0.19) | 0.803 |
| **Bathrooms** |  | 0.74 | (0.45, | 1.22) | 0.238 |  | 0.24 | (-0.08, | 0.56) | 0.138 |
| **Living areas^2^** |  | 1.41 | (0.75, | 2.63) | 0.284 |  | 0.03 | (-0.35, | 0.41) | 0.872 |
|  |  |  |  |  |  |  |  |  |  |  |
| **Type of toilet** |  |  |  |  |  |  |  |  |  |  |
| **None, bucket or hanging latrine** | | 1.00 |  |  |  |  | 1.00 |  |  |  |
| **Bore hole toilet^3^** |  | 0.40 | (0.13, | 1.28) | 0.124 |  | 0.48 | (-0.48, | 1.44) | 0.325 |
| **Pour flush toilet** |  | 0.29 | (0.10, | 0.87) | 0.026 |  | 0.82 | (-0.09, | 1.73) | 0.078 |
| **Flush toilet with septic tank** | | 0.30 | (0.10, | 0.87) | 0.026 |  | 0.87 | (-0.04, | 1.77) | 0.062 |
| **Flush toilet connected with sewerage system** | | 0.34 | (0.11, | 1.00) | 0.049 |  | 0.86 | (-0.05, | 1.77) | 0.063 |
|  |  |  |  |  |  |  |  |  |  |  |
| **Toilet shared with other household (versus not shared)** | | 2.43 | (1.47, | 4.02) | 0.001 |  | -0.71 | (-1.38, | -0.03) | 0.040 |
|  |  |  |  |  |  |  |  |  |  |  |
| **Main source of drinking water** | | | |  |  |  |  |  |  |  |
| **Unprotected source^4^** |  | 1.00 |  |  |  |  | 1.00 |  |  |  |
| **Public standpipe or other protected source^5^** | | 0.83 | (0.20, | 3.52) | 0.803 |  | 0.31 | (-1.26, | 1.89) | 0.697 |
| **Piped into yard** |  | 0.41 | (0.09, | 1.76) | 0.228 |  | 0.85 | (-0.72, | 2.41) | 0.291 |
| **Piped into house** |  | 0.47 | (0.12, | 1.89) | 0.286 |  | 0.63 | (-0.92, | 2.18) | 0.424 |
|  |  |  |  |  |  |  |  |  |  |  |
| **Main method of garbage disposal** | | | | |  |  |  |  |  |  |
| **Buried, burned or thrown** | | 1.00 |  |  |  |  | 1.00 |  |  |  |
| **Collected and thrown for recycling** | | 0.95 | (0.67, | 1.33) | 0.763 |  | 0.05 | (-0.16, | 0.25) | 0.659 |
| **Collected irregularly by local authority** | | 1.21 | (0.78, | 1.89) | 0.397 |  | -0.12 | (-0.39, | 0.15) | 0.371 |
| **Collected regularly by local authority** | | 1.29 | (1.04, | 1.59) | 0.018 |  | -0.21 | (-0.34, | -0.09) | 0.001 |
|  |  |  |  |  |  |  |  |  |  |  |
| Estimates based on mixed-effects Poisson regression models adjusted for all other variables above, and for clustering at the household level. | | | | | | | | | | |
| ^1^Stunting or height-for-age and BMI-for-age were classified using the WHO 2007 reference. ^2^Living areas include dining rooms but not kitchens; ^3^Bore hole toilet both with or without cover; ^4^Unprotected sources: unprotected dug well, or water taken directly from pond or stream; ^5^Protected sources: protected dug well or spring, or water from bottles or tanker truck. | | | | | | | | | | |

| **Table S8. Sensitivity analysis: effect of consideration of maternal age on relative risk of stunting^1^ associated with sociodemographic indices (N=3337).** | | | | | | | | | | |
| --- | --- | --- | --- | --- | --- | --- | --- | --- | --- | --- |
|  |  | **Maternal age excluded (main analysis)** | | | |  | **Maternal age included** | | | |
|  |  | **Risk ratio (95% confidence interval)** | | | ***P*** |  | **Risk ratio (95% confidence interval)** | | | ***P*** |
|  |  |  |  |  |  |  |  |  |  |  |
| **Female sex (versus male)** | | 1.07 | (0.94, | 1.21) | 0.318 |  | 1.07 | (0.94, | 1.21) | 0.317 |
|  |  |  |  |  |  |  |  |  |  |  |
| **Age** |  | 1.08 | (1.06, | 1.10) | <0.001 |  | 1.09 | (1.06, | 1.11) | <0.001 |
|  |  |  |  |  |  |  |  |  |  |  |
| **Ethnicity** |  |  |  |  |  |  |  |  |  |  |
| **Malay** |  | 1.00 |  |  |  |  | 1.00 |  |  |  |
| **Indian** |  | 0.65 | (0.51, | 0.84) | 0.001 |  | 0.65 | (0.50, | 0.84) | 0.001 |
| **Chinese** |  | 0.62 | (0.50, | 0.78) | <0.001 |  | 0.62 | (0.50, | 0.77) | 0.000 |
| **Bumiputera/Orang Asli** |  | 0.98 | (0.59, | 1.64) | 0.951 |  | 0.97 | (0.58, | 1.62) | 0.917 |
| **Other** |  | 1.01 | (0.54, | 1.91) | 0.963 |  | 1.01 | (0.54, | 1.90) | 0.970 |
|  |  |  |  |  |  |  |  |  |  |  |
| **BMI-for-age^1^ status** |  |  |  |  |  |  |  |  |  |  |
| **Underweight** |  | 1.17 | (0.96, | 1.44) | 0.123 |  | 1.18 | (0.96, | 1.44) | 0.117 |
| **Normal** |  | 1.00 |  |  |  |  | 1.00 |  |  |  |
| **Overweight or obese** |  | 0.79 | (0.67, | 0.93) | 0.005 |  | 0.79 | (0.67, | 0.93) | 0.005 |
|  |  |  |  |  |  |  |  |  |  |  |
| **Birth order** |  |  |  |  |  |  |  |  |  |  |
| **1** |  | 1.00 |  |  |  |  | 1.00 |  |  |  |
| **2** |  | 1.05 | (0.89, | 1.22) | 0.579 |  | 1.05 | (0.90, | 1.23) | 0.528 |
| **3** |  | 1.03 | (0.83, | 1.27) | 0.809 |  | 1.04 | (0.84, | 1.29) | 0.715 |
| **4+** |  | 1.17 | (0.90, | 1.53) | 0.243 |  | 1.20 | (0.91, | 1.57) | 0.190 |
|  |  |  |  |  |  |  |  |  |  |  |
| **Maternal height (cm) category** | | | |  |  |  |  |  |  |  |
| **160 +** |  | 1.00 |  |  |  |  | 1.00 |  |  |  |
| **155 - 159** |  | 1.00 | (0.81, | 1.24) | 0.991 |  | 1.00 | (0.81, | 1.24) | 0.988 |
| **150 - 154** |  | 1.02 | (0.83, | 1.25) | 0.861 |  | 1.02 | (0.84, | 1.25) | 0.824 |
| **145-149** |  | 1.45 | (1.18, | 1.79) | <0.001 |  | 1.47 | (1.19, | 1.81) | <0.001 |
| **< 145** |  | 1.62 | (1.24, | 2.11) | <0.001 |  | 1.66 | (1.27, | 2.18) | <0.001 |
|  |  |  |  |  |  |  |  |  |  |  |
| **Maternal current underweight (versus BMI ≥ 18.5)** | | 0.94 | (0.60, | 1.47) | 0.794 |  | 0.94 | (0.60, | 1.47) | 0.790 |
|  |  |  |  |  |  |  |  |  |  |  |
| **Rooms per household member** | | | |  |  |  |  |  |  |  |
| **Bedrooms** |  | 0.90 | (0.65, | 1.25) | 0.533 |  | 0.93 | (0.67, | 1.30) | 0.683 |
| **Bathrooms** |  | 0.78 | (0.49, | 1.24) | 0.289 |  | 0.80 | (0.50, | 1.27) | 0.342 |
| **Living areas^2^** |  | 1.46 | (0.86, | 2.48) | 0.157 |  | 1.46 | (0.86, | 2.48) | 0.159 |
|  |  |  |  |  |  |  |  |  |  |  |
| **Type of toilet** |  |  |  |  |  |  |  |  |  |  |
| **None, bucket or hanging latrine** | | 1.00 |  |  |  |  | 1.00 |  |  |  |
| **Bore hole toilet^3^** |  | 0.91 | (0.28, | 3.03) | 0.884 |  | 0.90 | (0.27, | 2.97) | 0.858 |
| **Pour flush toilet** |  | 0.65 | (0.21, | 2.03) | 0.455 |  | 0.64 | (0.20, | 2.01) | 0.444 |
| **Flush toilet with septic tank** | | 0.61 | (0.20, | 1.92) | 0.403 |  | 0.61 | (0.19, | 1.90) | 0.392 |
| **Flush toilet connected with sewerage system** | | 0.76 | (0.24, | 2.38) | 0.634 |  | 0.75 | (0.24, | 2.35) | 0.618 |
|  |  |  |  |  |  |  |  |  |  |  |
| **Toilet shared with other household (versus not shared)** | | 1.69 | (0.83, | 3.41) | 0.145 |  | 1.69 | (0.84, | 3.42) | 0.143 |
|  |  |  |  |  |  |  |  |  |  |  |
| **Main source of drinking water** | | | |  |  |  |  |  |  |  |
| **Unprotected source^4^** |  | 1.00 |  |  |  |  | 1.00 |  |  |  |
| **Public standpipe or other protected source^5^** | | 0.45 | (0.06, | 3.37) | 0.436 |  | 0.44 | (0.06, | 3.33) | 0.430 |
| **Piped into yard** |  | 0.24 | (0.03, | 1.85) | 0.172 |  | 0.24 | (0.03, | 1.84) | 0.172 |
| **Piped into house** |  | 0.31 | (0.04, | 2.29) | 0.253 |  | 0.31 | (0.04, | 2.27) | 0.250 |
|  |  |  |  |  |  |  |  |  |  |  |
| **Main method of garbage disposal** | | | |  |  |  |  |  |  |  |
| **Buried, burned or thrown** | | 1.00 |  |  |  |  | 1.00 |  |  |  |
| **Collected and thrown for recycling** | | 1.06 | (0.84, | 1.34) | 0.603 |  | 1.15 | (0.88, | 1.51) | 0.313 |
| **Collected irregularly by local authority** | | 1.39 | (1.02, | 1.89) | 0.036 |  | 1.39 | (0.97, | 2.01) | 0.073 |
| **Collected regularly by local authority** | | 1.22 | (1.05, | 1.41) | 0.011 |  | 1.22 | (1.03, | 1.44) | 0.020 |
|  |  |  |  |  |  |  |  |  |  |  |
| **Maternal age** |  | N/A | | | |  | 0.99 | (0.98, | 1.01) | 0.306 |
|  |  |  |  |  |  |  |  |  |  |  |
| Estimates based on mixed-effects Poisson regression models adjusted for all other variables above, and for clustering at the household level. | | | | | | | | | | |
| ^1^Stunting and BMI-for-age were classified using the CDC 2000 reference. ^2^Living areas include dining rooms but not kitchens; ^3^Bore hole toilet both with or without cover; ^4^Unprotected sources: unprotected dug well, or water taken directly from pond or stream; ^5^Protected sources: protected dug well or spring, or water from bottles or tanker truck. | | | | | | | | | | |

| **Table S9. Sensitivity analysis: effect of consideration of head of household's, maternal, and paternal education on risk of stunting^1^ associated with sociodemographic indices.** | | | | | | | | | | | | | | | | | | | | |
| --- | --- | --- | --- | --- | --- | --- | --- | --- | --- | --- | --- | --- | --- | --- | --- | --- | --- | --- | --- | --- |
|  |  | **No education (N = 3791)** | | | |  | **Head of household education (N = 3782)** | | | |  | **Maternal education (N = 3625)** | | | |  | **Paternal education (N = 2704)** | | | |
|  |  | **Risk ratio (95% confidence interval)** | | | ***P*** |  | **Risk ratio (95% confidence interval)** | | | ***P*** |  | **Risk ratio (95% confidence interval)** | | | ***P*** |  | **Risk ratio (95% confidence interval)** | | | ***P*** |
|  |  |  |  |  |  |  |  |  |  |  |  |  |  |  |  |  |  |  |  |  |
| **Female sex (versus male)** |  | 1.10 | (0.98, | 1.24) | 0.117 |  | 1.11 | (0.98, | 1.25) | 0.103 |  | 1.08 | (0.96, | 1.22) | 0.215 |  | 1.11 | (0.97, | 1.28) | 0.138 |
|  |  |  |  |  |  |  |  |  |  |  |  |  |  |  |  |  |  |  |  |  |
| **Age** |  | 1.08 | (1.06, | 1.10) | <0.001 |  | 1.07 | (1.05, | 1.09) | <0.001 |  | 1.08 | (1.06, | 1.10) | <0.001 |  | 1.07 | (1.05, | 1.10) | <0.001 |
|  |  |  |  |  |  |  |  |  |  |  |  |  |  |  |  |  |  |  |  |  |
| **Ethnicity** |  |  |  |  |  |  |  |  |  |  |  |  |  |  |  |  |  |  |  |  |
| **Malay** |  | 1.00 |  |  |  |  | 1.00 |  |  |  |  | 1.00 |  |  |  |  | 1.00 |  |  |  |
| **Indian** |  | 0.66 | (0.52, | 0.84) | 0.001 |  | 0.67 | (0.52, | 0.85) | 0.001 |  | 0.67 | (0.52, | 0.86) | 0.001 |  | 0.65 | (0.50, | 0.86) | 0.002 |
| **Chinese** |  | 0.68 | (0.56, | 0.83) | <0.001 |  | 0.69 | (0.56, | 0.84) | <0.001 |  | 0.67 | (0.55, | 0.82) | <0.001 |  | 0.66 | (0.52, | 0.84) | 0.001 |
| **Bumiputera/Orang Asli** |  | 0.96 | (0.59, | 1.56) | 0.857 |  | 0.99 | (0.60, | 1.66) | 0.984 |  | 1.03 | (0.59, | 1.79) | 0.915 |  | 0.89 | (0.41, | 1.96) | 0.775 |
| **Other** |  | 0.91 | (0.48, | 1.70) | 0.756 |  | 0.92 | (0.49, | 1.73) | 0.802 |  | 0.93 | (0.49, | 1.75) | 0.827 |  | 0.63 | (0.26, | 1.52) | 0.303 |
|  |  |  |  |  |  |  |  |  |  |  |  |  |  |  |  |  |  |  |  |  |
| **BMI-for-age^1^ status** |  |  |  |  |  |  |  |  |  |  |  |  |  |  |  |  |  |  |  |  |
| **Underweight** |  | 1.19 | (0.99, | 1.43) | 0.068 |  | 1.18 | (0.98, | 1.42) | 0.082 |  | 1.20 | (0.99, | 1.45) | 0.059 |  | 1.09 | (0.87, | 1.37) | 0.460 |
| **Normal** |  | 1.00 |  |  |  |  | 1.00 |  |  |  |  | 1.00 |  |  |  |  | 1.00 |  |  |  |
| **Overweight or obese** |  | 0.79 | (0.67, | 0.92) | 0.003 |  | 0.79 | (0.68, | 0.92) | 0.003 |  | 0.79 | (0.67, | 0.92) | 0.003 |  | 0.78 | (0.65, | 0.93) | 0.007 |
|  |  |  |  |  |  |  |  |  |  |  |  |  |  |  |  |  |  |  |  |  |
| **Birth order** |  |  |  |  |  |  |  |  |  |  |  |  |  |  |  |  |  |  |  |  |
| **1** |  | 1.00 |  |  |  |  | 1.00 |  |  |  |  | 1.00 |  |  |  |  | 1.00 |  |  |  |
| **2** |  | 1.03 | (0.89, | 1.19) | 0.718 |  | 1.02 | (0.89, | 1.18) | 0.752 |  | 1.03 | (0.89, | 1.20) | 0.689 |  | 1.04 | (0.88, | 1.23) | 0.661 |
| **3** |  | 0.99 | (0.82, | 1.21) | 0.947 |  | 0.99 | (0.81, | 1.20) | 0.893 |  | 0.99 | (0.81, | 1.21) | 0.899 |  | 0.97 | (0.77, | 1.22) | 0.774 |
| **4+** |  | 1.05 | (0.82, | 1.34) | 0.728 |  | 1.03 | (0.81, | 1.33) | 0.795 |  | 1.04 | (0.80, | 1.34) | 0.765 |  | 1.12 | (0.85, | 1.49) | 0.424 |
|  |  |  |  |  |  |  |  |  |  |  |  |  |  |  |  |  |  |  |  |  |
| **Maternal height (cm) category** |  |  |  |  |  |  |  |  |  |  |  |  |  |  |  |  |  |  |  |  |
| **160 +** |  | 1.00 |  |  |  |  | 1.00 |  |  |  |  | 1.00 |  |  |  |  | 1.00 |  |  |  |
| **155 - 159** |  | 1.03 | (0.84, | 1.27) | 0.759 |  | 1.04 | (0.84, | 1.28) | 0.718 |  | 1.07 | (0.86, | 1.33) | 0.537 |  | 1.12 | (0.89, | 1.42) | 0.342 |
| **150 - 154** |  | 1.06 | (0.87, | 1.29) | 0.580 |  | 1.07 | (0.88, | 1.30) | 0.522 |  | 1.10 | (0.90, | 1.35) | 0.354 |  | 1.14 | (0.91, | 1.43) | 0.260 |
| **145-149** |  | 1.44 | (1.18, | 1.76) | <0.001 |  | 1.46 | (1.19, | 1.79) | <0.001 |  | 1.54 | (1.25, | 1.90) | <0.001 |  | 1.53 | (1.21, | 1.94) | <0.001 |
| **< 145** |  | 1.53 | (1.20, | 1.94) | 0.001 |  | 1.55 | (1.22, | 1.97) | <0.001 |  | 1.61 | (1.24, | 2.08) | <0.001 |  | 1.79 | (1.33, | 2.40) | <0.001 |
|  |  |  |  |  |  |  |  |  |  |  |  |  |  |  |  |  |  |  |  |  |
| **Maternal current underweight (versus BMI ≥ 18.5)** | | 0.86 | (0.56, | 1.30) | 0.463 |  | 0.85 | (0.56, | 1.29) | 0.453 |  | 0.95 | (0.62, | 1.44) | 0.801 |  | 0.80 | (0.49, | 1.32) | 0.387 |
|  |  |  |  |  |  |  |  |  |  |  |  |  |  |  |  |  |  |  |  |  |
| **Rooms per household member** |  |  |  |  |  |  |  |  |  |  |  |  |  |  |  |  |  |  |  |  |
| **Bedrooms** |  | 1.04 | (0.77, | 1.40) | 0.808 |  | 1.03 | (0.76, | 1.39) | 0.832 |  | 1.07 | (0.79, | 1.46) | 0.653 |  | 1.00 | (0.69, | 1.45) | 0.996 |
| **Bathrooms** |  | 0.67 | (0.44, | 1.04) | 0.074 |  | 0.69 | (0.44, | 1.06) | 0.090 |  | 0.68 | (0.43, | 1.06) | 0.090 |  | 0.76 | (0.45, | 1.26) | 0.284 |
| **Living areas^2^** |  | 1.16 | (0.70, | 1.92) | 0.553 |  | 1.20 | (0.73, | 1.98) | 0.477 |  | 1.09 | (0.65, | 1.82) | 0.752 |  | 1.16 | (0.63, | 2.11) | 0.635 |
|  |  |  |  |  |  |  |  |  |  |  |  |  |  |  |  |  |  |  |  |  |
| **Type of toilet** |  |  |  |  |  |  |  |  |  |  |  |  |  |  |  |  |  |  |  |  |
| **None, bucket or hanging latrine** |  | 1.00 |  |  |  |  | 1.00 |  |  |  |  | 1.00 |  |  |  |  | 1.00 |  |  |  |
| **Bore hole toilet^3^** |  | 0.57 | (0.24, | 1.36) | 0.206 |  | 0.57 | (0.24, | 1.37) | 0.209 |  | 0.55 | (0.23, | 1.31) | 0.177 |  | 0.86 | (0.26, | 2.87) | 0.801 |
| **Pour flush toilet** |  | 0.41 | (0.18, | 0.92) | 0.030 |  | 0.41 | (0.18, | 0.93) | 0.032 |  | 0.41 | (0.18, | 0.93) | 0.033 |  | 0.68 | (0.22, | 2.14) | 0.511 |
| **Flush toilet with septic tank** |  | 0.38 | (0.17, | 0.87) | 0.021 |  | 0.39 | (0.17, | 0.87) | 0.022 |  | 0.38 | (0.17, | 0.86) | 0.021 |  | 0.64 | (0.21, | 2.02) | 0.452 |
| **Flush toilet connected with sewerage system** |  | 0.46 | (0.20, | 1.04) | 0.062 |  | 0.46 | (0.20, | 1.04) | 0.062 |  | 0.47 | (0.21, | 1.05) | 0.066 |  | 0.84 | (0.27, | 2.64) | 0.763 |
|  |  |  |  |  |  |  |  |  |  |  |  |  |  |  |  |  |  |  |  |  |
| **Toilet shared with other household (versus not shared)** | | 1.72 | (0.88, | 3.32) | 0.110 |  | 1.73 | (0.89, | 3.35) | 0.105 |  | 1.65 | (0.85, | 3.20) | 0.140 |  | 1.70 | (0.84, | 3.45) | 0.140 |
|  |  |  |  |  |  |  |  |  |  |  |  |  |  |  |  |  |  |  |  |  |
| **Main source of drinking water** |  |  |  |  |  |  |  |  |  |  |  |  |  |  |  |  |  |  |  |  |
| **Unprotected source^4^** |  | 1.00 |  |  |  |  | 1.00 |  |  |  |  | 1.00 |  |  |  |  | 1.00 |  |  |  |
| **Public standpipe or other protected source^5^** |  | 0.52 | (0.13, | 2.18) | 0.375 |  | 0.53 | (0.13, | 2.22) | 0.385 |  | 0.48 | (0.11, | 2.05) | 0.325 |  | 0.46 | (0.10, | 2.05) | 0.308 |
| **Piped into yard** |  | 0.25 | (0.06, | 1.04) | 0.057 |  | 0.25 | (0.06, | 1.07) | 0.062 |  | 0.23 | (0.05, | 0.98) | 0.047 |  | 0.21 | (0.05, | 0.98) | 0.047 |
| **Piped into house** |  | 0.35 | (0.09, | 1.41) | 0.140 |  | 0.36 | (0.09, | 1.44) | 0.147 |  | 0.32 | (0.08, | 1.31) | 0.113 |  | 0.32 | (0.08, | 1.38) | 0.128 |
|  |  |  |  |  |  |  |  |  |  |  |  |  |  |  |  |  |  |  |  |  |
| **Main method of garbage disposal** |  |  |  |  |  |  |  |  |  |  |  |  |  |  |  |  |  |  |  |  |
| **Buried, burned or thrown** |  | 1.00 |  |  |  |  | 1.00 |  |  |  |  | 1.00 |  |  |  |  | 1.00 |  |  |  |
| **Collected and thrown for recycling** |  | 1.06 | (0.82, | 1.39) | 0.646 |  | 1.08 | (0.83, | 1.40) | 0.582 |  | 1.06 | (0.81, | 1.38) | 0.666 |  | 1.03 | (0.76, | 1.40) | 0.851 |
| **Collected irregularly by local authority** |  | 1.39 | (1.00, | 1.92) | 0.047 |  | 1.37 | (0.99, | 1.89) | 0.056 |  | 1.42 | (1.03, | 1.97) | 0.034 |  | 1.39 | (0.96, | 2.00) | 0.081 |
| **Collected regularly by local authority** |  | 1.22 | (1.04, | 1.42) | 0.014 |  | 1.22 | (1.04, | 1.42) | 0.014 |  | 1.23 | (1.04, | 1.44) | 0.013 |  | 1.19 | (0.99, | 1.43) | 0.067 |
|  |  |  |  |  |  |  |  |  |  |  |  |  |  |  |  |  |  |  |  |  |
| **Head of household/maternal/paternal education** | | |  |  |  |  |  |  |  |  |  |  |  |  |  |  |  |  |  |  |
| **None** |  |  |  |  |  |  | 1.00 |  |  |  |  | 1.00 |  |  |  |  | 1.00 |  |  |  |
| **Primary** |  |  |  |  |  |  | 0.52 | (0.12, | 2.17) | 0.369 |  | 1.04 | (0.70, | 1.54) | 0.865 |  | 1.25 | (0.52, | 3.00) | 0.625 |
| **Secondary** |  |  |  |  |  |  | 0.64 | (0.16, | 2.62) | 0.532 |  | 1.15 | (0.78, | 1.70) | 0.477 |  | 1.47 | (0.61, | 3.55) | 0.387 |
| **Tertiary/Diploma** |  |  |  |  |  |  | 0.67 | (0.16, | 2.77) | 0.581 |  | 1.14 | (0.69, | 1.87) | 0.611 |  | 1.95 | (0.78, | 4.89) | 0.153 |
|  |  |  |  |  |  |  |  |  |  |  |  |  |  |  |  |  |  |  |  |  |
| Estimates based on Poisson regression models adjusted for all other variables above. Models were not adjusted for clustering at the household level in order to facilitate convergence. | | | | | | | | | | | | | | | | | | | | |
| ^1^Stunting and BMI-for-age were classified using the CDC 2000 reference. ^2^Living areas include dining rooms but not kitchens; ^3^Bore hole toilet both with or without cover; ^4^Unprotected sources: unprotected dug well, or water taken directly from pond or stream; ^5^Protected sources: protected dug well or spring, or water from bottles or tanker truck. | | | | | | | | | | | | | | | | | | | | |

| **Table S10. Sensitivity analysis: effect of consideration of maternal and paternal height on risk of stunting^1^ associated with sociodemographic indices (I).** | | | | | | | | | | | | | | | | | | | | |
| --- | --- | --- | --- | --- | --- | --- | --- | --- | --- | --- | --- | --- | --- | --- | --- | --- | --- | --- | --- | --- |
|  |  | **Maternal height (N = 3791)** | | | | | | | | |  | **Paternal height (N = 2719)** | | | | | | | | |
|  |  | **Excluding maternal height** | | | |  | **Including maternal height** | | | |  | **Excluding paternal height** | | | |  | **Including paternal height** | | | |
|  |  | **Risk ratio (95% confidence interval)** | | | ***P*** |  | **Risk ratio (95% confidence interval)** | | | ***P*** |  | **Risk ratio (95% confidence interval)** | | | ***P*** |  | **Risk ratio (95% confidence interval)** | | | ***P*** |
|  |  |  |  |  |  |  |  |  |  |  |  |  |  |  |  |  |  |  |  |  |
| **Female sex (versus male)** | | 1.11 | (1.00, | 1.22) | 0.049 |  | 1.10 | (1.00, | 1.22) | 0.054 |  | 1.10 | (0.98, | 1.24) | 0.090 |  | 1.09 | (0.97, | 1.22) | 0.139 |
|  |  |  |  |  |  |  |  |  |  |  |  |  |  |  |  |  |  |  |  |  |
| **Age** |  | 1.07 | (1.06, | 1.09) | <0.001 |  | 1.08 | (1.06, | 1.09) | <0.001 |  | 1.07 | (1.05, | 1.09) | <0.001 |  | 1.07 | (1.05, | 1.09) | <0.001 |
|  |  |  |  |  |  |  |  |  |  |  |  |  |  |  |  |  |  |  |  |  |
| **Ethnicity** |  |  |  |  |  |  |  |  |  |  |  |  |  |  |  |  |  |  |  |  |
| **Malay** |  | 1.00 |  |  |  |  | 1.00 |  |  |  |  | 1.00 |  |  |  |  | 1.00 |  |  |  |
| **Indian** |  | 0.64 | (0.51, | 0.81) | <0.001 |  | 0.65 | (0.51, | 0.82) | <0.001 |  | 0.63 | (0.48, | 0.82) | 0.001 |  | 0.64 | (0.49, | 0.84) | 0.001 |
| **Chinese** |  | 0.66 | (0.54, | 0.81) | <0.001 |  | 0.67 | (0.54, | 0.82) | <0.001 |  | 0.62 | (0.48, | 0.79) | <0.001 |  | 0.62 | (0.48, | 0.79) | <0.001 |
| **Bumiputera/Orang Asli** | | 1.06 | (0.71, | 1.58) | 0.782 |  | 0.97 | (0.66, | 1.43) | 0.886 |  | 0.72 | (0.39, | 1.34) | 0.301 |  | 0.68 | (0.37, | 1.23) | 0.203 |
| **Other** |  | 0.90 | (0.41, | 1.96) | 0.793 |  | 0.90 | (0.43, | 1.91) | 0.792 |  | 0.64 | (0.26, | 1.54) | 0.320 |  | 0.63 | (0.26, | 1.53) | 0.309 |
|  |  |  |  |  |  |  |  |  |  |  |  |  |  |  |  |  |  |  |  |  |
| **BMI-for-age^1^ status** | | |  |  |  |  |  |  |  |  |  |  |  |  |  |  |  |  |  |  |
| **Underweight** |  | 1.19 | (1.02, | 1.40) | 0.028 |  | 1.19 | (1.02, | 1.39) | 0.029 |  | 1.09 | (0.90, | 1.33) | 0.369 |  | 1.10 | (0.91, | 1.33) | 0.343 |
| **Normal** |  | 1.00 |  |  |  |  | 1.00 |  |  |  |  | 1.00 |  |  |  |  | 1.00 |  |  |  |
| **Overweight or obese** | | 0.78 | (0.68, | 0.90) | <0.001 |  | 0.78 | (0.68, | 0.90) | 0.001 |  | 0.80 | (0.69, | 0.94) | 0.008 |  | 0.80 | (0.69, | 0.94) | 0.007 |
|  |  |  |  |  |  |  |  |  |  |  |  |  |  |  |  |  |  |  |  |  |
| **Birth order** |  |  |  |  |  |  |  |  |  |  |  |  |  |  |  |  |  |  |  |  |
| **1** |  | 1.00 |  |  |  |  | 1.00 |  |  |  |  | 1.00 |  |  |  |  | 1.00 |  |  |  |
| **2** |  | 1.01 | (0.91, | 1.13) | 0.790 |  | 1.03 | (0.92, | 1.14) | 0.649 |  | 1.03 | (0.91, | 1.16) | 0.660 |  | 1.03 | (0.91, | 1.17) | 0.620 |
| **3** |  | 0.97 | (0.82, | 1.14) | 0.714 |  | 0.99 | (0.84, | 1.16) | 0.867 |  | 1.00 | (0.83, | 1.20) | 0.978 |  | 1.01 | (0.84, | 1.22) | 0.900 |
| **4+** |  | 1.01 | (0.80, | 1.26) | 0.956 |  | 1.03 | (0.82, | 1.29) | 0.809 |  | 1.06 | (0.83, | 1.36) | 0.628 |  | 1.07 | (0.83, | 1.37) | 0.596 |
|  |  |  |  |  |  |  |  |  |  |  |  |  |  |  |  |  |  |  |  |  |
| **Maternal or paternal height** | | N/A | | | |  | 0.98 | (0.97, | 0.99) | <0.001 |  | N/A | | | |  | 0.98 | (0.97, | 0.99) | <0.001 |
|  |  |  |  |  |  |  |  |  |  |  |  |  |  |  |  |  |  |  |  |  |
| **Maternal current underweight (versus BMI ≥ 18.5)** | | 0.85 | (0.56, | 1.28) | 0.436 |  | 0.86 | (0.57, | 1.30) | 0.484 |  | 0.80 | (0.48, | 1.34) | 0.399 |  | 0.82 | (0.50, | 1.35) | 0.435 |
|  |  |  |  |  |  |  |  |  |  |  |  |  |  |  |  |  |  |  |  |  |
| **Rooms per household member** | | | | |  |  |  |  |  |  |  |  |  |  |  |  |  |  |  |  |
| **Bedrooms** |  | 1.08 | (0.82, | 1.41) | 0.599 |  | 1.04 | (0.80, | 1.36) | 0.779 |  | 1.01 | (0.72, | 1.43) | 0.949 |  | 1.00 | (0.72, | 1.40) | 0.988 |
| **Bathrooms** |  | 0.68 | (0.47, | 1.00) | 0.051 |  | 0.68 | (0.47, | 0.99) | 0.042 |  | 0.73 | (0.46, | 1.16) | 0.188 |  | 0.75 | (0.48, | 1.17) | 0.208 |
| **Living areas^2^** |  | 1.08 | (0.67, | 1.72) | 0.759 |  | 1.16 | (0.73, | 1.85) | 0.530 |  | 1.13 | (0.64, | 1.98) | 0.669 |  | 1.03 | (0.59, | 1.81) | 0.912 |
|  |  |  |  |  |  |  |  |  |  |  |  |  |  |  |  |  |  |  |  |  |
| **Type of toilet** |  |  |  |  |  |  |  |  |  |  |  |  |  |  |  |  |  |  |  |  |
| **None, bucket or hanging latrine** | | 1.00 |  |  |  |  | 1.00 |  |  |  |  | 1.00 |  |  |  |  | 1.00 |  |  |  |
| **Bore hole toilet^3^** | | 0.55 | (0.26, | 1.15) | 0.112 |  | 0.56 | (0.25, | 1.22) | 0.146 |  | 0.82 | (0.47, | 1.42) | 0.478 |  | 0.82 | (0.48, | 1.41) | 0.474 |
| **Pour flush toilet** |  | 0.41 | (0.20, | 0.81) | 0.011 |  | 0.40 | (0.19, | 0.85) | 0.017 |  | 0.68 | (0.45, | 1.04) | 0.074 |  | 0.70 | (0.46, | 1.05) | 0.087 |
| **Flush toilet with septic tank** | | 0.38 | (0.19, | 0.76) | 0.006 |  | 0.39 | (0.18, | 0.81) | 0.012 |  | 0.62 | (0.41, | 0.94) | 0.025 |  | 0.65 | (0.43, | 0.98) | 0.038 |
| **Flush toilet connected with sewerage system** | | 0.45 | (0.22, | 0.89) | 0.023 |  | 0.46 | (0.22, | 0.96) | 0.040 |  | 0.83 | (0.54, | 1.26) | 0.376 |  | 0.87 | (0.57, | 1.33) | 0.520 |
|  |  |  |  |  |  |  |  |  |  |  |  |  |  |  |  |  |  |  |  |  |
| **Toilet shared with other household (versus not shared)** | | 1.86 | (1.15, | 2.98) | 0.011 |  | 1.75 | (1.09, | 2.80) | 0.020 |  | 1.92 | (1.14, | 3.22) | 0.014 |  | 1.96 | (1.19, | 3.22) | 0.008 |
|  |  |  |  |  |  |  |  |  |  |  |  |  |  |  |  |  |  |  |  |  |
| **Main source of drinking water** | | | | |  |  |  |  |  |  |  |  |  |  |  |  |  |  |  |  |
| **Unprotected source^4^** | | 1.00 |  |  |  |  | 1.00 |  |  |  |  | 1.00 |  |  |  |  | 1.00 |  |  |  |
| **Public standpipe or other protected source^5^** | | 0.47 | (0.32, | 0.67) | <0.001 |  | 0.56 | (0.40, | 0.78) | 0.001 |  | 0.52 | (0.32, | 0.84) | 0.008 |  | 0.59 | (0.37, | 0.94) | 0.026 |
| **Piped into yard** |  | 0.23 | (0.14, | 0.37) | <0.001 |  | 0.27 | (0.17, | 0.43) | <0.001 |  | 0.24 | (0.14, | 0.43) | <0.001 |  | 0.26 | (0.15, | 0.44) | <0.001 |
| **Piped into house** | | 0.32 | (0.26, | 0.40) | <0.001 |  | 0.38 | (0.32, | 0.43) | <0.001 |  | 0.37 | (0.27, | 0.51) | <0.001 |  | 0.40 | (0.29, | 0.55) | <0.001 |
|  |  |  |  |  |  |  |  |  |  |  |  |  |  |  |  |  |  |  |  |  |
| **Main method of garbage disposal** | | | | | |  |  |  |  |  |  |  |  |  |  |  |  |  |  |  |
| **Buried, burned or thrown** | | 1.00 |  |  |  |  | 1.00 |  |  |  |  | 1.00 |  |  |  |  | 1.00 |  |  |  |
| **Collected and thrown for recycling** | | 1.06 | (0.84, | 1.35) | 0.608 |  | 1.05 | (0.83, | 1.32) | 0.688 |  | 1.03 | (0.78, | 1.37) | 0.825 |  | 1.00 | (0.75, | 1.33) | 0.990 |
| **Collected irregularly by local authority** | | 1.37 | (1.02, | 1.84) | 0.039 |  | 1.38 | (1.01, | 1.87) | 0.042 |  | 1.50 | (1.07, | 2.10) | 0.020 |  | 1.39 | (1.01, | 1.92) | 0.044 |
| **Collected regularly by local authority** | | 1.20 | (1.03, | 1.40) | 0.018 |  | 1.23 | (1.06, | 1.43) | 0.007 |  | 1.21 | (1.01, | 1.45) | 0.038 |  | 1.22 | (1.02, | 1.46) | 0.026 |
|  |  |  |  |  |  |  |  |  |  |  |  |  |  |  |  |  |  |  |  |  |
| Estimates based on mixed-effects Poisson regression models adjusted for all other variables above, and for clustering at the household level. | | | | | | | | | | | | | | | | | | | | |
| ^1^Stunting and BMI-for-age were classified using the CDC 2000 reference. ^2^Living areas include dining rooms but not kitchens; ^3^Bore hole toilet both with or without cover; ^4^Unprotected sources: unprotected dug well, or water taken directly from pond or stream; ^5^Protected sources: protected dug well or spring, or water from bottles or tanker truck. | | | | | | | | | | | | | | | | | | | | |

| **Table S11. Sensitivity analysis: effect of consideration of maternal and paternal height on risk of stunting^1^ associated with sociodemographic indices (II).** | | | | | | | | | | | | | | | | | | | | |
| --- | --- | --- | --- | --- | --- | --- | --- | --- | --- | --- | --- | --- | --- | --- | --- | --- | --- | --- | --- | --- |
|  |  | **Maternal height (N = 3791)** | | | |  | **Maternal height (N = 2719)** | | | |  | **Paternal height (N = 2719)** | | | |  | **Maternal + paternal height (N = 2719)** | | | |
|  |  | **Risk ratio (95% confidence interval)** | | | ***P*** |  | **Risk ratio (95% confidence interval)** | | | ***P*** |  | **Risk ratio (95% confidence interval)** | | | ***P*** |  | **Risk ratio (95% confidence interval)** | | | ***P*** |
|  |  |  |  |  |  |  |  |  |  |  |  |  |  |  |  |  |  |  |  |  |
| **Female sex (versus male)** | | 1.10 | (1.00, | 1.22) | 0.054 |  | 1.10 | (0.95, | 1.26) | 0.195 |  | 1.09 | (0.95, | 1.25) | 0.226 |  | 1.09 | (0.95, | 1.25) | 0.240 |
|  |  |  |  |  |  |  |  |  |  |  |  |  |  |  |  |  |  |  |  |  |
| **Age** |  | 1.08 | (1.06, | 1.09) | <0.001 |  | 1.07 | (1.05, | 1.10) | <0.001 |  | 1.07 | (1.05, | 1.09) | <0.001 |  | 1.07 | (1.05, | 1.09) | <0.001 |
|  |  |  |  |  |  |  |  |  |  |  |  |  |  |  |  |  |  |  |  |  |
| **Ethnicity** |  |  |  |  |  |  |  |  |  |  |  |  |  |  |  |  |  |  |  |  |
| **Malay** |  | 1.00 |  |  |  |  | 1.00 |  |  |  |  | 1.00 |  |  |  |  | 1.00 |  |  |  |
| **Indian** |  | 0.65 | (0.51, | 0.82) | <0.001 |  | 0.62 | (0.47, | 0.81) | 0.001 |  | 0.64 | (0.49, | 0.85) | 0.002 |  | 0.64 | (0.48, | 0.83) | 0.001 |
| **Chinese** |  | 0.67 | (0.54, | 0.82) | <0.001 |  | 0.61 | (0.48, | 0.77) | <0.001 |  | 0.62 | (0.49, | 0.78) | <0.001 |  | 0.61 | (0.48, | 0.78) | <0.001 |
| **Bumiputera/Orang Asli** |  | 0.97 | (0.66, | 1.43) | 0.886 |  | 0.67 | (0.32, | 1.41) | 0.291 |  | 0.68 | (0.32, | 1.42) | 0.304 |  | 0.65 | (0.31, | 1.37) | 0.255 |
| **Other** |  | 0.90 | (0.43, | 1.91) | 0.792 |  | 0.62 | (0.26, | 1.51) | 0.292 |  | 0.63 | (0.26, | 1.54) | 0.311 |  | 0.62 | (0.26, | 1.51) | 0.293 |
|  |  |  |  |  |  |  |  |  |  |  |  |  |  |  |  |  |  |  |  |  |
| **BMI-for-age^1^ status** |  |  |  |  |  |  |  |  |  |  |  |  |  |  |  |  |  |  |  |  |
| **Underweight** |  | 1.19 | (1.02, | 1.39) | 0.029 |  | 1.09 | (0.87, | 1.37) | 0.431 |  | 1.10 | (0.88, | 1.37) | 0.421 |  | 1.10 | (0.88, | 1.38) | 0.415 |
| **Normal** |  | 1.00 |  |  |  |  | 1.00 |  |  |  |  | 1.00 |  |  |  |  | 1.00 |  |  |  |
| **Overweight or obese** |  | 0.78 | (0.68, | 0.90) | 0.001 |  | 0.80 | (0.67, | 0.96) | 0.014 |  | 0.80 | (0.67, | 0.96) | 0.016 |  | 0.80 | (0.67, | 0.96) | 0.015 |
|  |  |  |  |  |  |  |  |  |  |  |  |  |  |  |  |  |  |  |  |  |
| **Birth order** |  |  |  |  |  |  |  |  |  |  |  |  |  |  |  |  |  |  |  |  |
| **1** |  | 1.00 |  |  |  |  | 1.00 |  |  |  |  | 1.00 |  |  |  |  | 1.00 |  |  |  |
| **2** |  | 1.03 | (0.92, | 1.14) | 0.649 |  | 1.04 | (0.88, | 1.23) | 0.649 |  | 1.03 | (0.87, | 1.22) | 0.715 |  | 1.04 | (0.88, | 1.23) | 0.643 |
| **3** |  | 0.99 | (0.84, | 1.16) | 0.867 |  | 1.02 | (0.81, | 1.28) | 0.861 |  | 1.01 | (0.81, | 1.27) | 0.918 |  | 1.03 | (0.82, | 1.29) | 0.818 |
| **4+** |  | 1.03 | (0.82, | 1.29) | 0.809 |  | 1.10 | (0.83, | 1.45) | 0.518 |  | 1.07 | (0.81, | 1.41) | 0.640 |  | 1.09 | (0.83, | 1.45) | 0.532 |
|  |  |  |  |  |  |  |  |  |  |  |  |  |  |  |  |  |  |  |  |  |
| **Maternal height** |  | 0.98 | (0.97, | 0.99) | <0.001 |  | 0.98 | (0.97, | 0.99) | <0.001 |  | N/A | | | |  | 0.98 | (0.97, | 0.99) | 0.002 |
| **Paternal height** |  | N/A | | | |  | N/A | | | |  | 0.98 | (0.97, | 0.99) | <0.001 |  | 0.98 | (0.97, | 0.99) | 0.001 |
|  |  |  |  |  |  |  |  |  |  |  |  |  |  |  |  |  |  |  |  |  |
| **Maternal current underweight (versus BMI ≥ 18.5)** | | 0.86 | (0.57, | 1.30) | 0.484 |  | 0.80 | (0.47, | 1.37) | 0.421 |  | 0.82 | (0.48, | 1.40) | 0.472 |  | 0.82 | (0.48, | 1.40) | 0.465 |
|  |  |  |  |  |  |  |  |  |  |  |  |  |  |  |  |  |  |  |  |  |
| **Rooms per household member** | | | |  |  |  |  |  |  |  |  |  |  |  |  |  |  |  |  |  |
| **Bedrooms** |  | 1.04 | (0.80, | 1.36) | 0.779 |  | 0.96 | (0.66, | 1.39) | 0.825 |  | 1.00 | (0.69, | 1.45) | 0.989 |  | 0.96 | (0.67, | 1.39) | 0.845 |
| **Bathrooms** |  | 0.68 | (0.47, | 0.99) | 0.042 |  | 0.74 | (0.45, | 1.23) | 0.250 |  | 0.75 | (0.45, | 1.25) | 0.269 |  | 0.75 | (0.45, | 1.25) | 0.269 |
| **Living areas^2^** |  | 1.16 | (0.73, | 1.85) | 0.530 |  | 1.22 | (0.67, | 2.21) | 0.513 |  | 1.03 | (0.57, | 1.88) | 0.917 |  | 1.12 | (0.62, | 2.03) | 0.713 |
|  |  |  |  |  |  |  |  |  |  |  |  |  |  |  |  |  |  |  |  |  |
| **Type of toilet** |  |  |  |  |  |  |  |  |  |  |  |  |  |  |  |  |  |  |  |  |
| **None, bucket or hanging latrine** | | 1.00 |  |  |  |  | 1.00 |  |  |  |  | 1.00 |  |  |  |  | 1.00 |  |  |  |
| **Bore hole toilet^3^** |  | 0.56 | (0.25, | 1.22) | 0.146 |  | 0.85 | (0.25, | 2.86) | 0.796 |  | 0.82 | (0.24, | 2.74) | 0.747 |  | 0.84 | (0.25, | 2.82) | 0.781 |
| **Pour flush toilet** |  | 0.40 | (0.19, | 0.85) | 0.017 |  | 0.68 | (0.22, | 2.13) | 0.506 |  | 0.70 | (0.22, | 2.19) | 0.535 |  | 0.69 | (0.22, | 2.16) | 0.522 |
| **Flush toilet with septic tank** | | 0.39 | (0.18, | 0.81) | 0.012 |  | 0.64 | (0.20, | 2.01) | 0.447 |  | 0.65 | (0.21, | 2.03) | 0.458 |  | 0.66 | (0.21, | 2.06) | 0.471 |
| **Flush toilet connected with sewerage system** | | 0.46 | (0.22, | 0.96) | 0.040 |  | 0.87 | (0.28, | 2.73) | 0.808 |  | 0.87 | (0.28, | 2.74) | 0.814 |  | 0.89 | (0.28, | 2.81) | 0.844 |
|  |  |  |  |  |  |  |  |  |  |  |  |  |  |  |  |  |  |  |  |  |
| **Toilet shared with other household (versus not shared)** | | 1.75 | (1.09, | 2.80) | 0.020 |  | 1.75 | (0.87, | 3.55) | 0.118 |  | 1.96 | (0.97, | 3.96) | 0.061 |  | 1.83 | (0.90, | 3.69) | 0.094 |
|  |  |  |  |  |  |  |  |  |  |  |  |  |  |  |  |  |  |  |  |  |
| **Main source of drinking water** | | | |  |  |  |  |  |  |  |  |  |  |  |  |  |  |  |  |  |
| **Unprotected source^4^** |  | 1.00 |  |  |  |  | 1.00 |  |  |  |  | 1.00 |  |  |  |  | 1.00 |  |  |  |
| **Public standpipe or other protected source^5^** | | 0.56 | (0.40, | 0.78) | 0.001 |  | 0.55 | (0.07, | 4.11) | 0.557 |  | 0.59 | (0.08, | 4.41) | 0.604 |  | 0.59 | (0.08, | 4.44) | 0.608 |
| **Piped into yard** |  | 0.27 | (0.17, | 0.43) | <0.001 |  | 0.25 | (0.03, | 1.88) | 0.177 |  | 0.26 | (0.03, | 1.95) | 0.189 |  | 0.26 | (0.03, | 1.95) | 0.189 |
| **Piped into house** |  | 0.38 | (0.32, | 0.43) | <0.001 |  | 0.37 | (0.05, | 2.71) | 0.328 |  | 0.40 | (0.05, | 2.92) | 0.366 |  | 0.39 | (0.05, | 2.88) | 0.359 |
|  |  |  |  |  |  |  |  |  |  |  |  |  |  |  |  |  |  |  |  |  |
| **Main method of garbage disposal** | | | |  |  |  |  |  |  |  |  |  |  |  |  |  |  |  |  |  |
| **Buried, burned or thrown** | | 1.00 |  |  |  |  | 1.00 |  |  |  |  | 1.00 |  |  |  |  | 1.00 |  |  |  |
| **Collected and thrown for recycling** | | 1.05 | (0.83, | 1.32) | 0.688 |  | 1.00 | (0.73, | 1.36) | 0.990 |  | 1.00 | (0.73, | 1.36) | 0.991 |  | 0.98 | (0.72, | 1.34) | 0.905 |
| **Collected irregularly by local authority** | | 1.38 | (1.01, | 1.87) | 0.042 |  | 1.48 | (1.02, | 2.16) | 0.040 |  | 1.39 | (0.96, | 2.03) | 0.084 |  | 1.40 | (0.96, | 2.05) | 0.077 |
| **Collected regularly by local authority** | | 1.23 | (1.06, | 1.43) | 0.007 |  | 1.23 | (1.03, | 1.48) | 0.024 |  | 1.22 | (1.02, | 1.47) | 0.029 |  | 1.24 | (1.03, | 1.48) | 0.022 |
|  |  |  |  |  |  |  |  |  |  |  |  |  |  |  |  |  |  |  |  |  |
| Estimates based on mixed-effects Poisson regression models adjusted for all other variables above, and for clustering at the household level. | | | | | | | | | | | | | | | | | | | | |
| ^1^Stunting and BMI-for-age were classified using the CDC 2000 reference. ^2^Living areas include dining rooms but not kitchens; ^3^Bore hole toilet both with or without cover; ^4^Unprotected sources: unprotected dug well, or water taken directly from pond or stream; ^5^Protected sources: protected dug well or spring, or water from bottles or tanker truck. | | | | | | | | | | | | | | | | | | | | |

| **Table S12. Sensitivity analysis: relative risk of stunting^1^ associated with sociodemographic indices and theoretically uncorrelated variables (N = 3434).** | | | | | |
| --- | --- | --- | --- | --- | --- |
|  |  | **Risk ratio (95% confidence interval)** | | | ***P*** |
|  |  |  |  |  |  |
| **Orthogonal variables** |  |  |  |  |  |
| **Maternal heart rate** |  | 1.00 | (0.99, | 1.01) | 0.888 |
| **Maternal diastolic blood pressure** |  | 1.00 | (1.00, | 1.01) | 0.547 |
| **Household Streamyx internet** |  | 1.02 | (0.86, | 1.20) | 0.860 |
| **Motorcycles per household member** |  | 1.15 | (0.84, | 1.59) | 0.380 |
|  |  |  |  |  |  |
| **Female sex (versus male)** |  | 1.11 | (1.00, | 1.23) | 0.046 |
|  |  |  |  |  |  |
| **Age** |  | 1.08 | (1.06, | 1.10) | <0.001 |
|  |  |  |  |  |  |
| **Ethnicity** |  |  |  |  |  |
| **Malay** |  | 1.00 |  |  |  |
| **Indian** |  | 0.68 | (0.53, | 0.87) | 0.003 |
| **Chinese** |  | 0.65 | (0.52, | 0.81) | <0.001 |
| **Bumiputera/Orang Asli** |  | 0.93 | (0.61, | 1.43) | 0.741 |
| **Other** |  | 0.96 | (0.42, | 2.17) | 0.913 |
|  |  |  |  |  |  |
| **BMI-for-age^1^ status** |  |  |  |  |  |
| **Underweight** |  | 1.19 | (1.01, | 1.41) | 0.036 |
| **Normal** |  | 1.00 |  |  |  |
| **Overweight or obese** |  | 0.81 | (0.70, | 0.93) | 0.003 |
|  |  |  |  |  |  |
| **Birth order** |  |  |  |  |  |
| **1** |  | 1.00 |  |  |  |
| **2** |  | 1.02 | (0.91, | 1.15) | 0.720 |
| **3** |  | 1.01 | (0.85, | 1.21) | 0.868 |
| **4 +** |  | 1.11 | (0.88, | 1.41) | 0.378 |
|  |  |  |  |  |  |
| **Maternal height (cm) category** |  |  |  |  |  |
| **160 +** |  | 1.00 |  |  |  |
| **155 - 159** |  | 1.02 | (0.82, | 1.28) | 0.834 |
| **150 - 154** |  | 1.07 | (0.87, | 1.32) | 0.533 |
| **145-149** |  | 1.45 | (1.17, | 1.79) | 0.001 |
| **< 145** |  | 1.47 | (1.15, | 1.88) | 0.002 |
|  |  |  |  |  |  |
| **Maternal current underweight (versus BMI ≥ 18.5)** |  | 0.89 | (0.59, | 1.34) | 0.575 |
|  |  |  |  |  |  |
| **Rooms per household member** |  |  |  |  |  |
| **Bedrooms** |  | 1.11 | (0.85, | 1.46) | 0.432 |
| **Bathrooms** |  | 0.62 | (0.42, | 0.92) | 0.017 |
| **Living areas^2^** |  | 1.07 | (0.66, | 1.74) | 0.795 |
|  |  |  |  |  |  |
| **Type of toilet** |  |  |  |  |  |
| **None, bucket or hanging latrine** |  | 1.00 |  |  |  |
| **Bore hole toilet^3^** |  | 0.58 | (0.24, | 1.40) | 0.226 |
| **Pour flush toilet** |  | 0.39 | (0.17, | 0.91) | 0.030 |
| **Flush toilet with septic tank** |  | 0.37 | (0.16, | 0.85) | 0.020 |
| **Flush toilet connected with sewerage system** |  | 0.46 | (0.20, | 1.07) | 0.073 |
|  |  |  |  |  |  |
| **Toilet shared with other household (versus not shared)** |  | 1.33 | (0.78, | 2.28) | 0.289 |
|  |  |  |  |  |  |
| **Main source of drinking water** |  |  |  |  |  |
| **Unprotected source^4^** |  | 1.00 |  |  |  |
| **Public standpipe or other protected source^5^** |  | 0.47 | (0.33, | 0.66) | <0.001 |
| **Piped into yard** |  | 0.21 | (0.13, | 0.36) | <0.001 |
| **Piped into house** |  | 0.35 | (0.29, | 0.41) | <0.001 |
|  |  |  |  |  |  |
| **Main method of garbage disposal** |  |  |  |  |  |
| **Buried, burned or thrown** |  | 1.00 |  |  |  |
| **Collected and thrown for recycling** |  | 1.01 | (0.80, | 1.29) | 0.904 |
| **Collected irregularly by local authority** |  | 1.39 | (1.02, | 1.89) | 0.035 |
| **Collected regularly by local authority** |  | 1.19 | (1.02, | 1.39) | 0.029 |
|  |  |  |  |  |  |
| Estimates based on mixed-effects Poisson regression models adjusted for all other variables above, and for clustering at the household level. | | | | | |
| ^1^Stunting and BMI-for-age were classified using the CDC 2000 reference. ^2^Living areas include dining rooms but not kitchens; ^3^Bore hole toilet both with or without cover; ^4^Unprotected sources: unprotected dug well, or water taken directly from pond or stream; ^5^Protected sources: protected dug well or spring, or water from bottles or tanker truck. | | | | | |

| **Table S13. Crude prevalence of stunting across categories of number of risk factors.** | | | | | | | | | |
| --- | --- | --- | --- | --- | --- | --- | --- | --- | --- |
|  |  | **Overall** | | **Normal height** | | **Stunted^1^** | |  | ***P*** |
|  |  |  |  |  |  |  |  |  |  |
| **Number of risk factors^2^** |  |  |  |  |  |  |  |  |  |
| **0** |  | 1003 | (26.5) | 784 | (78.2) | 219 | (21.8) |  |  |
| **1** |  | 2275 | (60.0) | 1610 | (70.8) | 665 | (29.2) |  |  |
| **2** |  | 479 | (12.6) | 301 | (62.8) | 178 | (37.2) |  |  |
| **3** |  | 34 | (0.9) | 17 | (50.0) | 17 | (50.0) |  | <0.001 |
|  |  |  |  |  |  |  |  |  |  |
| Differences between categories by stunting status were compared using Pearson's Chi squared test. | | | | | | | | | |
| ^1^Stunting and BMI-for-age were classified using the CDC 2000 reference. ^2^Risk factors include any of the following: Malay ethnicity, child underweight, maternal height less than 145 cm, living in a household with unprotected drinking water source (unprotected dug well, or water taken directly from pond or stream), living in household with no bucket or hanging latrine, and living in a household with a shared toilet. | | | | | | | | | |

| **Table S14. Relative risk of stunting^1^ associated with co-occurring risk factors.** | | | | | |
| --- | --- | --- | --- | --- | --- |
|  |  | **Risk ratio (95% confidence interval)** | | | ***P*** |
|  |  |  |  |  |  |
| **Number of risk factors^2^** |  |  |  |  |  |
| **0** |  | 1.00 |  |  |  |
| **1** |  | 1.43 | (1.22, | 1.69) | <0.001 |
| **2** |  | 1.84 | (1.50, | 2.27) | <0.001 |
| **3** |  | 2.48 | (1.51, | 4.09) | <0.001 |
|  |  |  |  |  |  |
| **Female sex (versus male)** |  | 1.12 | (0.99, | 1.26) | 0.074 |
|  |  |  |  |  |  |
| **Age** |  | 1.08 | (1.06, | 1.09) | 0.000 |
|  |  |  |  |  |  |
| **Birth order** |  |  |  |  |  |
| **1** |  | 1.00 |  |  |  |
| **2** |  | 1.02 | (0.88, | 1.18) | 0.774 |
| **3** |  | 0.97 | (0.80, | 1.18) | 0.753 |
| **4+** |  | 0.99 | (0.77, | 1.26) | 0.906 |
|  |  |  |  |  |  |
| **Maternal current underweight (versus BMI ≥ 18.5)** | | 0.87 | (0.57, | 1.31) | 0.501 |
|  |  |  |  |  |  |
| **Rooms per household member** |  |  |  |  |  |
| **Bedrooms** |  | 1.12 | (0.84, | 1.49) | 0.452 |
| **Bathrooms** |  | 0.66 | (0.43, | 1.01) | 0.055 |
| **Living areas^3^** |  | 1.00 | (0.61, | 1.65) | 0.994 |
|  |  |  |  |  |  |
| **Main method of garbage disposal** |  |  |  |  |  |
| **Buried, burned or thrown** |  | 1.00 |  |  |  |
| **Collected and thrown for recycling** |  | 1.03 | (0.80, | 1.34) | 0.800 |
| **Collected irregularly by local authority** |  | 1.24 | (0.90, | 1.71) | 0.180 |
| **Collected regularly by local authority** |  | 1.13 | (0.98, | 1.31) | 0.087 |
|  |  |  |  |  |  |
| Estimates based on mixed-effects Poisson regression models adjusted for all other variables above, and for clustering at the household level. | | | | | |
| ^1^Stunting and BMI-for-age were classified using the CDC 2000 reference. ^2^Risk factors include any of the following: Malay ethnicity, child underweight, maternal height less than 145 cm, living in a household with unprotected drinking water source (unprotected dug well, or water taken directly from pond or stream), living in household with no bucket or hanging latrine, and living in a household with a shared toilet. ^3^Living areas include dining rooms but not kitchens. | | | | | |
| *P* for likelihood ratio test comparing this model with an identical model including number of risk factors as a continuous covariate: 0.704. In the continuous model, the linear increase in risk of stunting with each additional risk factor was 1.35 (95% confidence interval: 1.23, 1.49, P < 0.001). | | | | | |
